# Emission and Transcriptional Regulation of Aroma Variation in *Oncidium* Twinkle ‘Red Fantasy’ Under Diel Rhythm

**DOI:** 10.3390/plants13223232

**Published:** 2024-11-17

**Authors:** Yan Chen, Shengyuan Zhong, Lan Kong, Ronghui Fan, Yan Xu, Yiquan Chen, Huaiqin Zhong

**Affiliations:** 1 Institute of Crop Sciences, Fujian Academy of Agricultural Sciences (Fujian Germplasm Resources Center), Fuzhou 350013, China; spencer_cy@163.com (Y.C.);; 2 Fujian Engineering Research Center for Characteristic Floriculture, Fuzhou 350013, China; 3 Fuzhou Sub-Center for New Plant Variety Tests, Ministry of Agriculture and Rural Affairs, Fuzhou 350013, China; 4 College of Landscape Architecture and Art, Fujian Agriculture and Forestry University, Fuzhou 350002, China

**Keywords:** *Oncidium hybridum*, floral scent, diel rhythm, transcriptome, light/dark treatment

## Abstract

*Oncidium hybridum* is one of the important cut-flowers in the world. However, the lack of aroma in its cut-flower varieties greatly limits the sustainable development of the *Oncidium hybridum* cut-flowers industry. This paper is an integral investigation of the diel pattern and influencing factors of the aroma release of *Oncidium* Twinkle ‘Red Fantasy’. GC-MS analysis revealed that the release of 3-Carene peaked at 10:00, while Butyl tiglate and Prenyl senecioate did so at 14:00, with a diel rhythm. By analyzing the correlation network between aroma component synthesis and differentially expressed genes, 15 key structural genes were detected and regulated by multiple circadian rhythm-related transcription factors. *Cluster-17371.18_TPS*, *Cluster-65495.1_TPS*, *Cluster-46699.0_TPS*, *Cluster-60935.10_DXS*, *Cluster-47205.4_IDI*, and *Cluster-65313.7_LOX* were key genes in the terpenoid and fatty acid derivative biosynthetic pathway, which were co-expressed with aroma release. Constant light/dark treatments revealed that the diurnal release of 3-Carene may be influenced by light and the circadian clock, and Butyl tiglate and Prenyl senecioate may be mainly determined by endogenous circadian clock. Under constant light treatment, the *TPS*, *DXS*, *IDI*, and *LOX* genes seem to lose their regulatory role in the release of aroma compounds from *Oncidium* Twinkle ‘Red Fantasy’. Under constant dark treatment, the *TPS* genes were consistent with the release pattern of 3-Carene, which may be a key factor in regulating the diel rhythm of 3-Carene biosynthesis. These results laid a theoretical foundation for the study of floral transcriptional regulation and genetic engineering technology breeding of *Oncidium hybridum*.

## 1. Introduction

Floral scent is one of the important qualities and ornamental traits of ornamental plants, serving as a medium for communication between plant flowers and the external environment. It plays a crucial role in attracting pollinators, resisting pathogens and parasites, and serving as relevant signals in response to biotic and abiotic stress [1]. Meanwhile, floral volatiles are widely used in perfumes, scents, and cosmetics. Floral scent consists of a mixture of volatile organic compounds (VOCs), which are classified into terpenoids, phenylpropanoids/benzenoids, fatty acid derivatives, and amino acid derivatives according to biosynthetic pathways [2]. Terpenes are the most diverse and abundant secondary metabolites in plant floral scents, synthesized through two independent pathways: the mevalonic acid (MVA) pathway and the methylerythritol phosphate (MEP) pathway [3,4]. The MEP pathway mainly synthesizes sesquiterpenes, monoterpenes, and diterpenes, while the MVA pathway mainly synthesizes sesquiterpenes [5]. Among them, the MEP pathway uses pyruvate and glyceraldehyde 3-phosphate (G3P) as precursors, and undergoes seven enzymatic reactions to produce isopentenyl diphosphate (IPP) or dimethylallyl diphosphate (DMAPP) [6,7,8]. IPP and DMAPP generate sesquiterpenes, monoterpenes, diterpenes, and volatile carotenoid derivatives under the catalysis of geranylgeranyl diphosphate synthase (GGPPS), geranyl diphosphate synthase (GPPS), and terpene synthase (TPS) enzymes [6,7,8]. Fatty acid derivatives are another major class of VOCs in plants, and their biosynthesis mainly occurs through the lipoxygenase (LOX) pathway [9]. Linoleic and linolenic acid are the main substrates of LOX in plant tissues, which catalyze the production of unsaturated 9-hydroperoxides or 13-hydroperoxides through 9- and 13-LOX [5,10]. Unsaturated 9-hydroperoxides or 13-hydroperoxides are further oxidized and converted to volatile compounds by hydroperoxide lyase (HPL) and alcohol dehydrogenase (ADH) [5,10]. Up to now, there have been a lot of studies on the function, biosynthesis, and regulation of VOCs in ornamental plants [5,11]. However, the composition and abundance of VOCs in floral scents vary greatly among plants [12]. 

The synthesis of floral compounds in plants is a dynamic process throughout the entire plant cycle and is regulated by many factors, including light [13], flowering stages [14], diurnal patterns [15], and visiting insects [16]. The release of flower compounds usually exhibits a distinct diel rhythm, which has been demonstrated in many plants. These include snapdragon [17], rose [18], and Wisteria [19] flowers that release scent in the daytime, as well as *Petunia axillaris* [20] and *Nymphaea* subg. *Hydrocallis* [21] that release scent in the night. The release of the main active aroma compounds Myrcene and (E)-β-ocimene in snapdragon exhibited a diel rhythm variation with an increase during the day and a decrease at night [17]. The emission of the four major volatiles (E)-β-ocimene, benzaldehyde, geranyl isovalerate, and benzyl tiglate in *Globe amaranth* showed a diel rhythmic pattern, with the maximum emission occurring in the late afternoon and the lowest emission occurring at night [22]. The main floral components of *Petunia axillaris* were methyl benzoate, benzaldehyde, and isoeugenol, which exhibited a circadian rhythm [20]. The highest release occurred at 0 a.m. and lowest at 12 p.m. [20]. The release of floral compounds is regulated by genes, and the expression of many genes in the MEP pathway of terpenoid synthesis is rhythmic [17]. The monoterpene synthetase and deoxyoxyketose-5-phosphate synthetase (*DXPS*) genes of snapdragon showed a consistent change pattern of terpenoids, and this circadian rhythm was regulated by the endogenous circadian clock [17]. The expression levels of *DXS*, *DXR2*, *MCT*, and *HDR* genes in *Populus trichocarpa* followed a consistent rhythm with the release of monoterpenes [23]. The expression of terpene synthase gene *OfTPS5* in osmanthus flower showed a rhythm of increasing in the day and decreasing at night [24]. Many esters’ metabolism was also regulated by the circadian clock. In *Rosa hybrida*, the release of geranyl acetate exhibited a consistent diel rhythm with the gene expression of the *RhAAT* in the LOX pathway [18]. In addition, the accumulation of *ZmLOX10* in the maize LOX pathway was strictly regulated by the diel rhythm, and was consistent with the highest photosynthetic activity over time [25]. At present, the research on the molecular mechanism of diel rhythm mainly focuses on Arabidopsis [26,27]. In petunias, the circadian rhythm-related gene *PhLHY* regulates the volatilization of aroma compounds by inhibiting the expression of genes on the floral volatile benzenoid/phenylpropanoid pathway during the day [28]. Yon et al. [29] silenced two key circadian rhythm-related genes, *NaLHY* and *NaZTL*, resulting in changes in the endogenous rhythm of volatile compound release. Many studies have found that genes involved in the synthesis and metabolism of different volatile compounds exhibit rhythmic expression. However, the specific metabolic mechanisms by which the diel rhythm affects floral scent are still unclear. 

*Oncidium hybridum* belongs to the genus *Oncidium* of the Orchidaceae, also known as “dancing lady orchid”, native to the Neotropical region. *Oncidium hybridum* is one of the most important cut-flower varieties in the world with its unique flower posture and shape, bright colors, long flowering period, and wide adaptability [30]. At present, there are few varieties of cut-flowers of *Oncidium hybridum* on the market, all of which are scentless varieties [30]. Floral scent is one of the key characteristics of many floricultural crops. Due to the poor affinity of interspecific and intergeneric hybridization, it is difficult to introduce aroma traits into cut-flower varieties by traditional breeding methods. This greatly limits the sustainable development of the cut-flower industry of *Oncidium hybridum* [31]. Therefore, a thorough analysis of the molecular mechanism underlying the scents formation of *Oncidium hybridum* is necessary to effectively regulate the breeding process of aroma varieties and improve breeding efficiency. At present, there are few aromatic varieties of *Oncidium hybridum*, and research on its aromatic volatile compounds has mainly focused on potted varieties such as *Oncidium* Sharry Baby [32,33] and *Oncidium* Rosy Sunset [34]. The characteristic aroma compounds of *Oncidium* Sharry Baby include 3,7-dimethyl-1,3,6-Octatriene, (E)-2-Butenoic acid, 2-(methylenecyclopropyl) prop-2-yl ester, 3,7-dimethyl-1,6-Octadien-3-ol, and (Z)-3,7-dimethyl-2,6-Octadien-1-ol, among which terpenes are important compounds affecting the aroma of *Oncidium* Sharry Baby [32,33]. Yeh et al. [35] validated the mechanism of the diel release of the scent of *Oncidium* Sharry Baby. It was also shown that *Circadian Clock Associated 1* (*CCA1*) transcription factor (TF) affected the diel rhythm release of scent by up-regulating *TPS* under constant light treatment. The main VOCs released by *Oncidium* Rosy Sunset was the Linalool belonging to terpenoid [34]. Its aroma release has a rhythmic pattern, with the highest concentration from 10:00 to 12:00, and the lowest concentration from 20:00 to 24:00 [34]. The *Oncidium* Twinkle series was known for its intense scent, but the type and rhythm regulation of its aroma have not been reported. Up to now, the biosynthesis of orchid scents is still unclear, and only a few studies have reported on this. Hsiao et al. [30] reported the monoterpenoid biosynthetic pathway and related genes in Phalaenopsis. The *PbGDPS* gene was specifically expressed in flowers, and aroma biosynthesis of Phalaenopsis was mainly due to the production of geranyl and Linalool by *DXPS* [30]. In *Cymbidium ensifolium*, the release of methyl jasmonate and its related genes *CeLOX*, *CeAOS*, *CeAOC*, and *CeJMT* showed the same trend in sepals and petals, and were all highly regulated [36]. These studies laid a foundation for exploring the formation and diel rhythm regulation mechanism of *Oncidium hybridum* scents. However, due to its long life cycle and large genome size, little was known about the way of producing its floral scents. 

In this study, the potted aroma variety *Oncidium* Twinkle ‘Red Fantasy’ was used as the material, which has a strong floral aroma. We explored the mechanism of aroma release and diel rhythm-related mechanisms of *Oncidium* Twinkle ‘Red Fantasy’ based on the scientific phenomenon of the rhythmic release of its scent. The volatiles released by the *Oncidium* Twinkle ‘Red Fantasy’ were collected at different time points within 48 h (under 12 h light/12 h dark) using the dynamic headspace technique and analyzed using gas chromatography-mass spectrometry (GC-MS). Combined with transcriptome sequencing, the genes and TFs related to VOCs biosynthesis and the diel rhythm pathway in the transcriptome of *Oncidium* Twinkle ‘Red Fantasy’ were analyzed and excavated. In order to further explore the relationship between aroma synthesis genes and the diel rhythm of aroma release, we detected the content of VOCs released and the expression of aroma synthesis genes in *Oncidium* Twinkle ‘Red Fantasy’ under constant light/dark treatment. These results will lay the foundation for analyzing the rhythmic release of aroma in *Oncidium hybridum*, and also provide a reference for the genetic engineering breeding of floral aroma. 

## 2. Results

### 2.1. Oncidium Twinkle ‘Red Fantasy’ VOCs Were Emitted by Diel Rhythm Pattern Synchronizing

**Figure 1:**
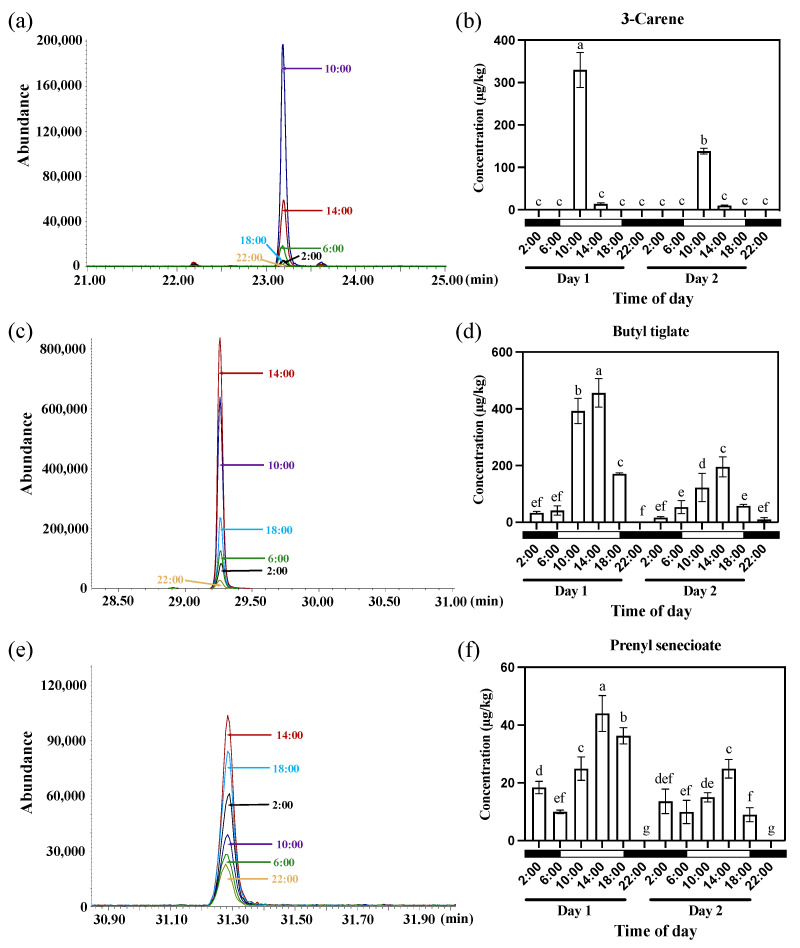
The emission patterns of three floral scent compounds—3-Carene, Butyl tiglate, and Prenyl senecioate—in *Oncidium* Twinkle ‘Red Fantasy’ under normal photoperiod (under 12 h light/12 h dark). (**a**) Overlapping analysis of 3-Carene ion current in samples at different time points within 24 h. The abscissa represents the retention time (min), and the ordinate represents the ion current intensity. (**b**) The emission patterns of 3-Carene from *Oncidium* Twinkle ‘Red Fantasy’ flowers within 48 h. (**c**) Overlapping analysis of Butyl tiglate ion current in samples at different time points within 24 h. The abscissa represents the retention time (min), and the ordinate represents the ion current intensity. (**d**) The emission patterns of Butyl tiglate from *Oncidium* Twinkle ‘Red Fantasy’ flowers within 48 h. (**e**) Overlapping analysis of Prenyl senecioate ion current in samples at different time points within 24 h. The abscissa represents the retention time (min), and the ordinate represents the ion current intensity. (**f**) The emission patterns of Prenyl senecioate from *Oncidium* Twinkle ‘Red Fantasy’ flowers within 48 h. Each treatment was conducted in triplicate with three technical repeats. Values are mean ± SD. Different lowercase letters indicate a statistically significant difference (*p* < 0.05).

To identify the diel rhythm pattern of VOCs associated with *Oncidium* Twinkle ‘Red Fantasy’, GC−MS was employed for profiling volatiles at different time points within 48 h (under 12 h light/12 h dark) of flowering. We collected samples every 4 h over two days. GC-MS analysis of the headspace trapping revealed five volatile components. Three typical peaks were detected in *Oncidium* Twinkle ‘Red Fantasy’ flowers and identified as 3-Carene, Butyl tiglate, and Prenyl senecioate, which accounted for >96% of the total peak areas (Figure S1). 3-Carene belongs to monoterpenes, and its release began to increase and peak around 10:00 (Days 1 and 2) during diel oscillation, followed by a rapid decrease (Figure 1a,b). Butyl tiglate and Prenyl senecioate belong to ester compounds, and their diel oscillation displayed a peak around 14:00 (Day 1 and 2) (Figure 1c–f). The emission of Butyl tiglate increased from 2:00 until 14:00, and decreased from 14:00 until 22:00 (Days 1 and 2) (Figure 1c,d). The emission of Prenyl senecioate decreased from 2:00 to 6:00, while it showed an upward trend from 6:00 to 14:00, and decreased from 14:00 to 22:00 (Days 1 and 2) (Figure 1e,f). The diel release pattern of three volatile components were almost the same across all two days (Figure 1). This indicated that the floral emission pattern of *Oncidium* Twinkle ‘Red Fantasy’ was a diel control that may be regulated by diel rhythmic genes. 

### 2.2. Overview of Transcriptome Sequencing

**Figure 2:**
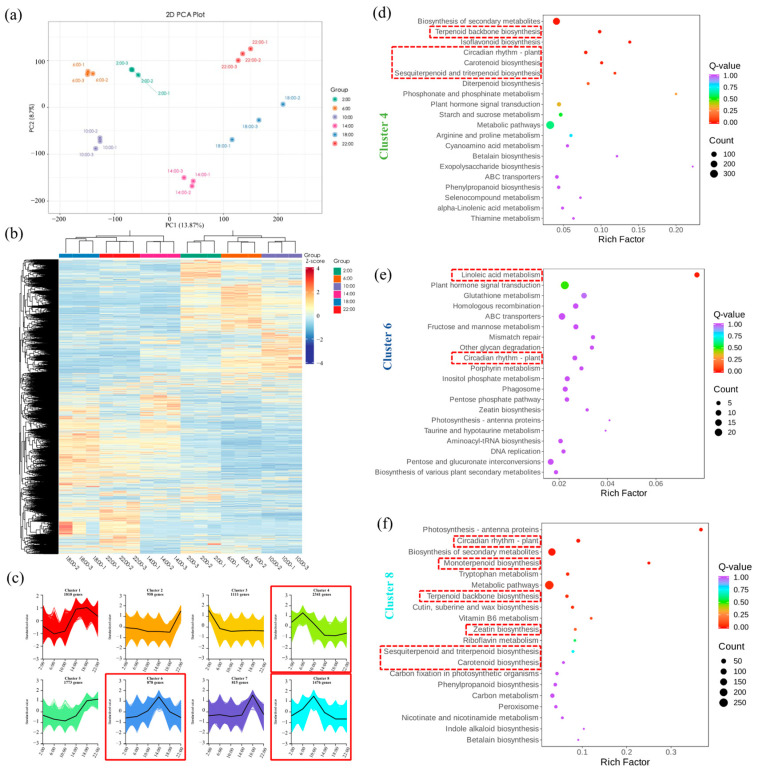
Transcriptomic analysis of *Oncidium* Twinkle ‘Red Fantasy’ at different time points within 24 h (under 12 h light/12 h dark). (**a**) Principal component analysis (PCA) plot showed overall differences among six groups (2:00, 6:00, 10:00, 14:00, 18:00, and 22:00) and the variability between intra-group samples. (**b**) Heatmap of differentially expressed genes (DEGs) sorted by K-means clustering across the samples collected at different time points. The numbers 1, 2, and 3 with each sample represented number of replicates. (**c**) Eight K-means clusters (Clusters 1–8) showed differential expression trends of DEGs at different time points. (**d**) KEGG enrichment analysis of DEGs in Cluster 4. The red boxes indicate metabolic pathways related to aroma rhythm release. (**e**) KEGG enrichment analysis of DEGs in Cluster 6. The red boxes indicate metabolic pathways related to aroma rhythm release. (**f**) KEGG enrichment analysis of DEGs in Cluster 8. The red boxes indicate metabolic pathways related to aroma rhythm release.

In order to obtain insights into the diel rhythm changes of floral VOCs biosynthesis of *Oncidium* Twinkle ‘Red Fantasy’, transcriptomic analysis at different time points within 24 h (under 12 h light/12 h dark) was performed. Principal component analysis (PCA) demonstrated that samples were well discriminated according to the classification of different time points, and clustered mainly according to three biological replicates (Figure 2a). The heatmap and hierarchical clustering showed different patterns of gene expression during the diel rhythm (from 2:00 to 22:00) (Figure 2b). Among them, the time points of ante meridiem (2:00, 6:00 and 10:00) and post meridiem (14:00, 18:00, and 22:00) were clustered, respectively. The results indicated that there were different gene expression patterns of *Oncidium* Twinkle ‘Red Fantasy’ between the ante meridiem and post meridiem.

To further explore the molecular regulatory mechanism of aroma formation under diel rhythm, RNA-seq data were analyzed by integrating aroma release patterns. We used the K-means clustering algorithm to classify the different characteristics of differentially expressed genes (DEGs). Eight different expression clusters (Cluster 1 to Cluster 8) were directly exhibited, with 1818, 958, 1111, 2341, 1773, 878, 815, and 1476 members, respectively (Figure 2c). Based on the change in the contents of key aroma compounds and gene expression profiles, we found that the gene expressions in Cluster 6 and Cluster 8 reached their peak at 14:00 and 10:00, respectively. This was similar to the trend of changes in the aroma content of *Oncidium* Twinkle ‘Red Fantasy’ (Figure 2c). In addition, the expression of genes regulating aroma synthesis may precede the release of aroma. Therefore, we focused on the functional annotation of genes in Cluster 4, Cluster 6, and Cluster 8 (Figure 2d). The content of 3-Carene peaked at 10:00, and the gene expression of Cluster 4 and Cluster 8 peaked at 6:00 and 10:00, respectively. The results of the Kyoto Encyclopedia of Genes and Genomes (KEGG) enrichment analysis showed that genes in Cluster 4 and Cluster 8 were significantly enriched in the pathways related to terpene synthesis, including “Terpenoid backbone biosynthesis”, “Carotenoid biosynthesis”, and “Sesquiterpenoid and triterpenoid biosynthesis”. In addition, Cluster 8 genes were also significantly enriched in “Monoterpenoid biosynthesis” and “Zeatin biosynthesis” pathways. The Cluster 6 genes were significantly enriched in the “Linoleic acid metabolism” pathway related to ester metabolism, following the same trend as the content of Butyl tiglate and Prenyl senecioate, which reached its peak at 14:00. Meanwhile, Cluster 4, Cluster 6, and Cluster 8 genes were enriched in the “Circadian rhythm-plant” pathway. Therefore, we speculated that the genes in Cluster 4 and Cluster 8 were closely related to terpenoid biosynthesis, and Cluster 6 genes were related to ester volatile compound biosynthesis. These genes may be the reason for the formation of diel rhythm changes in the aroma of *Oncidium* Twinkle ‘Red Fantasy’. 

### 2.3. Characterization of Fatty Acid Derivative and Terpenoid Biosynthetic Pathway Under Diel Rhythm 

**Figure 3:**
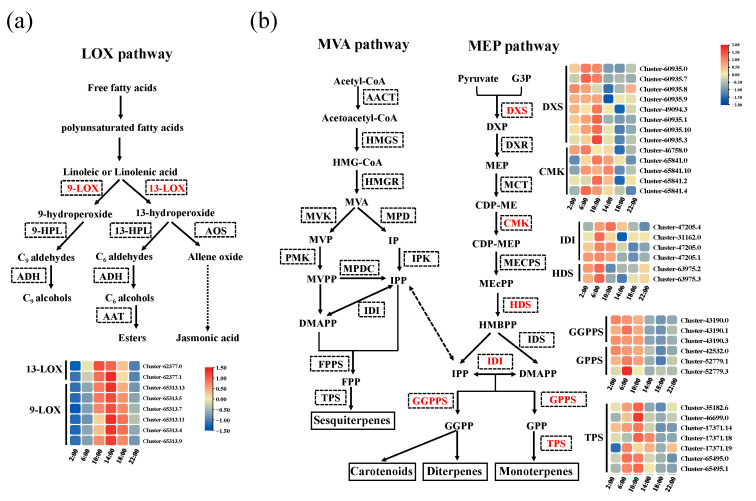
Overview of metabolites and DEGs in the biosynthesis pathways of fatty acid derivative and terpenoid in *Oncidium* Twinkle ‘Red Fantasy’. (**a**) The DEGs of Cluster 6 were enriched in the fatty acid derivative biosynthesis pathway. *9-lipoxygenase* (*9-LOX*), *13-lipoxygenase* (*13-LOX*), *9-hydroperoxide lyase* (*9-HPL*), *13-hydroperoxide lyase* (*13*-*HPL*), *alcohol dehydrogenase* (*ADH*), *allene oxide synthase* (*AOS*), and *alcohol acyltransferase* (*AAT*). The black dashed boxes represent genes enriched in the LOX pathway, and the red fonts represent differentially expressed genes. (**b**) The DEGs of Cluster 4 and Cluster 8 were enriched in the terpenoid biosynthesis pathway. *Acetyl*-*CoA acetyltransferase* (*AACT*), *hydroxymethylglutaryl*-*CoA synthase* (*HMGS*), *hydroxymethylglutaryl*-*CoA reductase* (*HMGR*), *mevalonate kinase* (*MVK*), *mevalonate phosphate decarboxylase* (*MPD*), *phosphomevalonate kinase* (*PMK*), *isopentenyl phosphate kinase* (*IPK*), *mevalonate diphosphate decarboxylase* (*MPDC*), *isopentenyl diphosphate isomerase* (*IDI*), *farnesyl pyrophosphate synthase* (*FPPS*), *terpenoid synthase* (*TPS*), *1-deoxy-D-xylulose 5-phosphate synthase* (*DXS*), *1-deoxy-D-xylulose 5-phosphate reductoisomerase* (*DXR*), *2-C-methyl-D-erythritol 4-phosphate cytidylyltransferase* (*MCT*), *4-(cytidine 5′-diphospho)-2-C-methyl-D-erythritol kinase* (*CMK*), *2-C-methyl-D-erythritol 2,4-cyclodiphosphate synthase* (*MECPS*), *4-hydroxy-3-methylbut-2-en-1-yl diphosphate synthase* (*HDS*), *isoprenyl diphosphate synthase* (*IDS*), *geranylgeranyl pyrophosphate synthase* (*GGPPS*), and *geranyl diphosphate synthase* (*GPPS*). The black dashed boxes represent genes enriched in the MVA and MEP pathway, and the red fonts represent differentially expressed genes.

To gain further insights into the biosynthesis of aroma compounds under diel rhythm, we outlined the biosynthesis pathways of fatty acid derivative and terpenoid based on the changes in the content of aroma compounds in *Oncidium* Twinkle ‘Red Fantasy’ and the KEGG database (Figure 3). Fatty acid derivatives are one of the main volatile organic compounds in plants, and lipoxygenase (LOX) was the crucial functional protein for the formation of the characteristic aroma compounds of the esters [9]. Polyunsaturated fatty acids are catabolized via the LOX pathway to form intermediates of 9-hydroperoxide and 13-hydroperoxide [5]. A total of 21 genes were found to be differentially expressed at 6:00 vs. 2:00, 10:00 vs. 2:00, 14:00 vs. 2:00, 18:00 vs. 2:00, 22:00 vs. 2:00, 10:00 vs. 6:00, 14:00 vs. 6:00, 18:00 vs. 6:00, 22:00 vs. 6:00, 14:00 vs. 10:00, 18:00 vs. 10:00, 22:00 vs. 10:00, 18:00 vs. 14:00, 22:00 vs. 14:00, and 22:00 vs. 18:00 at different steps of LOX pathway (Figure S2a). Among the 21 differentially enriched genes, the highly expressed genes were mainly concentrated at 14:00 (38.10%), while the remaining genes highly expressed at 2:00, 6:00, 10:00, and 18:00 were 9.52%, 23.80%, 14.29%, and 14.29%, respectively. Among them, eight *LOX* genes belong to Cluster 6, which was similar to the release diel rhythm of ester compounds in *Oncidium* Twinkle ‘Red Fantasy’, including two *13-LOX* genes (*Cluster-62377*.0 and *Cluster-62377.1*) and six *9-LOX* genes (*Cluster-65313.13*, *Cluster-65313.5*, *Cluster-65313.7*, *Cluster-65313.11*, *Cluster-65313.4*, and *Cluster-65313.9*) (Figure 3a). Consequently, it was reasonable to speculate that the eight differentially expressed *LOX* genes may lead to the peak emission of ester volatile compounds in *Oncidium* Twinkle ‘Red Fantasy’. 

In plants, volatile terpenes are derived from two distinct pathways, designated as the MEP pathway and the MVA pathway [11]. In this study, structural genes encoding enzymes involved in these two pathways were isolated, and their expression levels were evaluated at six time points within 24 h (2:00, 6:00, 10:00, 14:00, 18:00, and 22:00). In the MVA pathway and MEP pathway, a total of 46 structural genes related to terpenoid synthesis were differentially enriched (Figure S2b). Among the 46 differentially enriched genes, most of them had the highest expression at 6:00 (34.78%) or 10:00 (28.26%). The genes highly expressed at 2:00, 14:00, 18:00, and 22:00 accounted for 10.87%, 8.7%, 8.7%, and 6.52% of the total, respectively. The results showed that structural genes related to terpenoid synthesis were highly expressed in ante meridiem (2:00, 6:00, and 10:00). Based on the previous analysis, the genes in Cluster 4 and Cluster 8 were closely related to the biosynthesis of terpenoids. We identified 32 DEGs in Cluster 4 and Cluster 8 that were enriched in the MEP pathway, including *DXS* (*Cluster-60935.0*, *Cluster-60935.7*, *Cluster-60935.8*, *Cluster-60935.9*, *Cluster-49094.3*, *Cluster-60935.1*, *Cluster-60935.10* and *Cluster-60935.3*), *CMK* (*Cluster*-*46758*.0, *Cluster-65841.0*, *Cluster-65841.10*, *Cluster-65841.2* and *Cluster-65841.4*), *IDI* (*Cluster*-*31162*.0, *Cluster*-*47205.0*, *Cluster*-*47205.1* and *Cluster-47205.4*), *HDS* (*Cluster*-*63975.2* and *Cluster-63975.3*), *GGPPS* (*Cluster*-*43190.0*, *Cluster-43190.1* and *Cluster-43190.3*), *GPPS* (*Cluster*-*42532.0*, *Cluster-52779.1* and *Cluster-52779.3*) and *TPS* (*Cluster-35182.6*, *Cluster-46699.0*, *Cluster-17371.14*, *Cluster-17371.18*, *Cluster-17371.19*, *Cluster-65495.0*, and *Cluster-65495.1*) (Figure 3b). Taken together, Cluster 4 and Cluster 8 DEGs, enriched in the MEP pathway, may play an important role in the biosynthesis of dominant terpenoid. 

### 2.4. Fifteen Structural Genes and TF Genes Were Hub Genes Related to the Diel Rhythm of Aroma Synthesis in Oncidium Twinkle ‘Red Fantasy’

**Figure 4:**
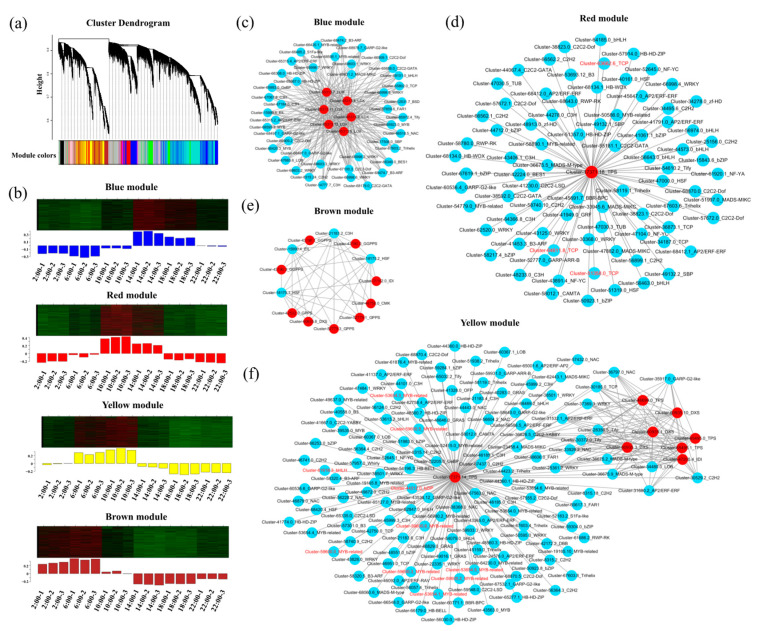
Establishment of weighted gene co-expression network analysis (WGCNA) modules of the differentially expressed genes (DEGs) at different time points. (**a**) Hierarchical clustering tree of the co-expression modules. The major tree branches constituted 10 distinct co-expression modules. (**b**) The gene expression patterns of the Blue, Red, Yellow, and Brown modules in WGCNA. The upper part was the clustering heatmap of genes within this module, with red indicating high expression and green indicating low expression. The lower part showed the expression patterns of module feature values in different samples. (**c**) Co-expression network of the genes from the Blue module. The red circles represent the key hub genes enriched in fatty acid derivative biosynthesis pathway, and the blue circles represent aroma synthesis related transcription factors (TFs). (**d**) Co-expression network of the genes from the Red module. The red circles represent the key hub genes enriched in terpenoid biosynthesis pathway, and the blue circles represent aroma synthesis related TFs. The red font represents TFs that were differentially enriched in the “Circadian rhythm-plant” pathway. (**e**) Co-expression network of the genes from the Brown module. The red circles represent the key hub genes enriched in terpenoid biosynthesis pathway, and the blue circles represent aroma synthesis related TFs. (**f**) Co-expression network of the genes from the Yellow module. The red circles represent the key hub genes enriched in terpenoid biosynthesis pathway, and the blue circles represent aroma synthesis related TFs. The red font represents TFs that were differentially enriched in the “Circadian rhythm-plant” pathway. The networks were visualized by Cytoscape (v3.5.1) software.

To explore co-expression networks of genes and possible relationships between the changes in diel rhythm of aroma synthesis and genes, we performed a weighted gene co-expression network analysis (WGCNA), which revealed 10 modules (labeled Blue, Turquoise, Black, Green, Pink, Purple, Brown, Yellow, Magenta, and Red) (Figure 4a,b). The expression trends in each module were shown in Figure 4b and Figure S3. We searched for highly connected TFs related to DEGs and biological processes of diel rhythm of aroma synthesis. Four modules were further analyzed, including the Blue, Red, Yellow, and Brown modules (Figure 4b). In the Blue module, genes were generally up-regulated from 2:00 to 14:00, and down-regulated from 14:00 to 22:00 (Figure 4b). Genes co-expressed in this module were significantly enriched in “Fatty acid degradation”, “α-Linolenic acid metabolism”, “Linolenic acid metabolism”, and “Fatty acid biosynthesis” (Figure S4a). In the Red module, genes were up-regulated from 2:00 to 10:00, and gradually down-regulated from 10:00 to 22:00 (Figure 4b). These genes were significantly enriched in “Circadian rhythm-plant” and “Monoterpenoid biosynthesis” (Figure S4b). In the Yellow module, genes were sharply up-regulated from 2:00 to 6:00, continuously up-regulated from 6:00 to 10:00, stayed at those levels, and then were down-regulated from 10:00 to 22:00 (Figure 4b). In the Brown module, genes were up-regulated from 2:00 to 6:00, and gradually down-regulated from 6:00 to 22:00 (Figure 4b). The Yellow and Brown module genes were significantly enriched in “Terpenoid backbone biosynthesis”, “Circadian rhythm-plant”, “Monoterpenoid biosynthesis”, “Carotenoid biosynthesis”, and “Zeatin biosynthesis”, which were closely associated with the synthesis of terpenes (Figure S4c). 

To identify highly connected TFs related to the diel rhythm of aroma synthesis in *Oncidium* Twinkle ‘Red Fantasy’, the selected modules were exported and visualized using Cytoscape (Figure 4c–e). Therefore, we discerned the TF genes from the network of Blue module, and a total of 44 TFs were identified and distributed across 25 families. Among them, *WRKY* genes with seven members presented the greatest number of TFs (Figure 4c). In the Red module, *Cluster-17371.18_TPS* was predicted to be regulated by 80 TFs, distributed across 25 families (Figure 4d). The nine structural genes in the Brown module were regulated by four transcription factors, including *Cluster-18175.1_HSF*, *Cluster*-*15990.4_EIL*, *Cluster-21183.2_C3H*, and *Cluster-18175.2_HSF* (Figure 4e). There were 243 pairs of TF-structural genes identified in the Yellow module, such as *MYB-related* (19), *C2H2* (11), *AP2/ERF* (9), *NAC* (9), and *WRKY* (9) (Figure 4f). These results suggest that TFs may participate in aroma-formation indirectly by modulating the expression of aroma-related structural genes. In the regulatory network of the four modules, we further screened TFs that were differentially enriched in the “Circadian rhythm-plant” pathway. In the Red module, three diel rhythm-related TFs *TCP* (*Cluster-63662.6_TCP, Cluster-64611.8_TCP,* and *Cluster-51358.0_TCP*) regulated *Cluster-17371.18_TPS* were identified, while none was identified in the Blue and Brown modules. Notably, we identified 10 diel rhythm-related TFs in the Yellow module, including eight *MYB-related* (*Cluster-53654.1_MYB-related, Cluster-53654.3_MYB-related, Cluster-53654.5_MYB-related, Cluster-59600.0_MYB-related, Cluster-59600.2_MYB-related, Cluster-59600.3_MYB-related, Cluster-59600.5_MYB-related,* and *Cluster-59600.6_MYB-related*), one *bHLH* (*Cluster-61618.3_bHLH*), and one *bZIP* (*Cluster-44312.0_bZIP*), all of which regulated the same structural gene (*Cluster-17371.14_TPS*) related to terpenoid synthesis. Therefore, it was speculated that *MYB*-related TFs and their related structural genes in the regulatory network were potential hub genes for the diel rhythm of aroma synthesis in *Oncidium* Twinkle ‘Red Fantasy’.

### 2.5. Expression Analysis of Aroma Synthesis Genes in Oncidium Twinkle ‘Red Fantasy’ Under Diel Rhythm

**Figure 5:**
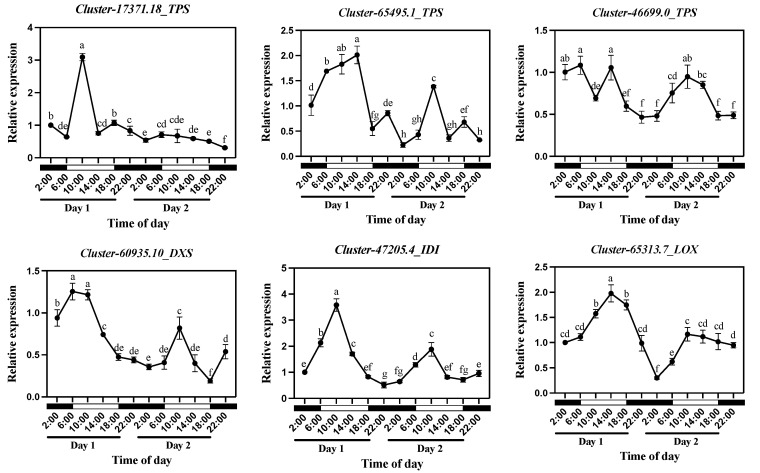
Relative expression of structural genes *Cluster-17371.18_TPS*, *Cluster-65495.1_TPS*, *Cluster-46699.0_TPS*, *Cluster-60935.10_DXS*, *Cluster*-*47205.4_IDI*, and *Cluster-65313.7_LOX* in *Oncidium* Twinkle ‘Red Fantasy’ flowers within 48 h (under 12 h light/12 h dark). Each treatment was conducted in triplicate with three technical repeats. Values are mean ± SD. Different lowercase letters indicate a statistically significant difference (*p* < 0.05).

To compare the transcription of aroma-related structural genes with the release patterns of VOCs under diel rhythm, the expression levels of six candidate structural genes (*Cluster-17371.18_TPS, Cluster-65495.1_TPS*, *Cluster-46699.0_TPS, Cluster-60935.10_DXS*, *Cluster-47205.4_IDI*, and *Cluster-65313.7_LOX*) (FPKM > 10) under diel rhythm were analyzed by quantitative real-time PCR (qRT-PCR) (Figure 5). Terpene synthases (TPS) constitute key enzymes in the biosynthetic pathway to produce monoterpene compounds. The GC–MS data suggested that the final product was 3-Carene (Figure 1). When we examined the expression patterns of *TPS* genes, the expression levels of *Cluster-17371.18_TPS* and *Cluster-65495.1_TPS* peaked at 10:00 (Figure 5). Among them, the expression of *Cluster-17371.18_TPS* was similar to the emission pattern of 3-Carene, mainly reflected on Day 1, while *Cluster-65495.1-TPS* showed similar peaks on both Day 1 and Day 2. However, *Cluster-46699.0_TPS* only showed a similar trend to 3-Carene on Day 2 (Figure 5). *DXS* and *IDI* genes are regarded as key rate-limiting enzymes in the MEP pathway, which directly affect the yield and efficiency of terpenoids [6,7,8]. The expression of *Cluster-60935.10_DXS* significantly increased from 2:00 to 6:00, maintained high expression from 6:00 to 10:00, and then significantly decreased (Day 1). At the same time, a peak was reached at 10:00 on Day 2 (Figure 5). Notably, *Cluster-47205.4_IDI* has a typical peak at 10:00 on both Day 1 and Day 2, which was consistent with the release trend of 3-Carene under diel rhythm (Figure 5). *Cluster-65313.7_LOX* was enriched in the fatty acid derivative biosynthesis pathway, and belonged to the Blue module in WGCNA analysis. During the diel oscillation, the expression of *Cluster-65313.7_LOX* was significantly up-regulated from 2:00 (Day 1) to 14:00 (Day 1), reaching its peak at 14:00 (Day 1), and then significantly down-regulated from 14:00 (Day 1) to 2:00 (Day 2). On Day 2, the expression level of *Cluster-65313.7_LOX* was significantly up-regulated from 2:00 to 10:00, and maintained high expression levels from 10:00 to 18:00 (Figure 5). The *Cluster-65313.7_LOX* expression trend was similar to the release pattern of Butyl tiglate and Prenyl senecioate in *Oncidium* Twinkle ‘Red Fantasy’, suggesting that *Cluster-65313.7_LOX* might be involved in the diel rhythmicity of ester volatile compound biosynthesis. In summary, the diel rhythm of the terpenoids in *Oncidium* Twinkle ‘Red Fantasy’ may be co-regulated by multiple enzymes in the synthetic pathway. 

### 2.6. Analysis of Aroma Release Pattern and Aroma Synthesis Genes Expression of Oncidium Twinkle ‘Red Fantasy’ Under Constant Light and Constant Dark Treatments

**Figure 6:**
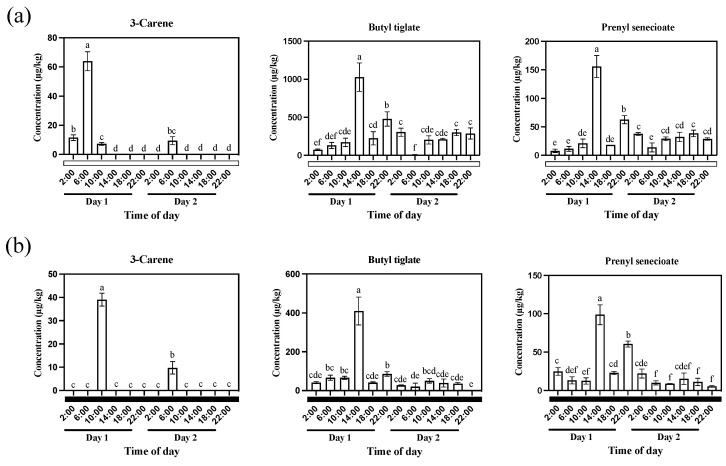
Analysis of aroma release pattern of *Oncidium* Twinkle ‘Red Fantasy’ under constant light and constant dark treatments. (**a**) The emission patterns of three floral scent compounds from *Oncidium* Twinkle ‘Red Fantasy’ flowers within 48 h under constant light. (**b**) The emission patterns of three floral scent compounds from *Oncidium* Twinkle ‘Red Fantasy’ flowers within 48 h under constant dark. Different lowercase letters indicate a statistically significant difference (*p* < 0.05).

To further explore the relationship between aroma synthesis genes and the diel rhythm of aroma release, we first examined the content of VOCs released under constant light and constant dark treatments (Figure 6). The GC-MS data showed that the release of 3-Carene reached its peak at 6:00 (Day 1) under constant light treatment, and a peak also appeared at the same time point (6:00) on Day 2 (Figure 6a). Interestingly, both ester compounds (Butyl tiglate, and Prenyl senecioate) showed the same tendency to release under constant light treatment. The content of Butyl tiglate and Prenyl senecioate gradually increased from 2:00 to 14:00 (Day 1), rapidly decreased at 18:00 (Day 1), and remained stable from 10:00 to 22:00 on Day 2 (Figure 6a). These results indicated that the aroma release of *Oncidium* Twinkle ‘Red Fantasy’ was not only influenced by diel rhythm, but also by light signals. Under constant dark treatment, 3-Carene showed peaks at 10:00 on Day 1 and 6:00 on Day 2 (Figure 6b). Notably, the change trends of Butyl tiglate and Prenyll senecioate contents were similar to those under constant light treatment, with a peak appearing at 14:00 (Day 1) (Figure 6b). Thus, the aroma release of *Oncidium* Twinkle ‘Red Fantasy’ may be more regulated by diel rhythms than light signals under a constant dark condition. 

**Figure 7:**
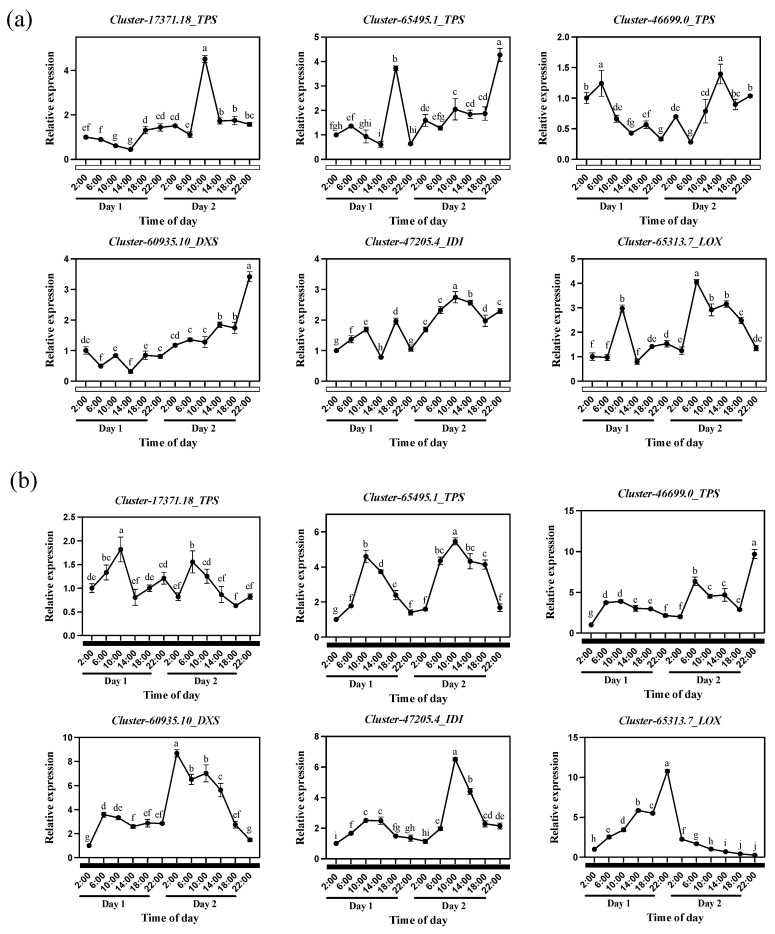
Analysis of aroma synthesis genes expression of *Oncidium* Twinkle ‘Red Fantasy’ under constant light and constant dark treatments. (**a**) Relative expression of structural genes *Cluster-17371.18_TPS, Cluster-65495.1_TPS*, *Cluster-46699.0_TPS, Cluster-60935.10_DXS*, *Cluster-47205.4_IDI*, and *Cluster-65313.7_LOX* in *Oncidium* Twinkle ‘Red Fantasy’ flowers within 48 h under constant light. (**b**) Relative expression of structural genes *Cluster-17371.18_TPS, Cluster-65495.1_TPS*, *Cluster-46699.0_TPS, Cluster-60935.10_DXS*, *Cluster-47205.4_IDI*, and *Cluster-65313.7_LOX* in *Oncidium* Twinkle ‘Red Fantasy’ flowers within 48 h under constant dark. Each treatment was conducted in triplicate with three technical repeats. Values are mean ± SD. Different lowercase letters indicate a statistically significant difference (*p* < 0.05).

When we explored the expression patterns of *TPS*, *DXS*, and *IDI* genes under constant light treatment, the expression of *TPS* genes (*Cluster-17371.18_TPS, Cluster-65495.1_TPS*, *Cluster-46699.0_TPS*) lost its rhythm and reached its peak at 10:00 (Day 2), 22:00 (Day 1), and 14:00 (Day 2), respectively (Figure 7a). The *IDI* gene was also in the same situation, and the expression reached its peak at 10:00. In response to the light signal, the expression of the *DXS* gene was gradually up-regulated with an increase in light duration from 14:00 (Day 1). For *Cluster-65313.7_LOX*, related to the biosynthesis of fatty acid derivatives, the expression peaks were mainly distributed at 10:00 (Day 1) and 6:00 (Day 2). Therefore, *TPS*, *DXS*, *IDI*, and *LOX* genes seem to lose their regulatory effect on the release of aroma compounds of *Oncidium* Twinkle ‘Red Fantasy’ under constant light treatment. We analyzed the expression of *TPS* genes under constant dark treatment and found that the expression trend of *Cluster-17371.18_TPS*, *Cluster-65495.1_TPS*, and *Cluster-46699.0_TPS* was similar to the release trend of 3-Carene (Figure 7b). It was up-regulated from 2:00 to 10:00 on Day 1, and from 2:00 to 6:00 on Day 2. The expression level of *Cluster-60935.10_DXS* was the highest at 6:00 on Day 1 and at 2:00 on Day 2, respectively, while the expression level of *Cluster-47205.4_IDI* was the highest at 10:00 (Day 1 and Day 2). The expression of *Cluster-65313.7_LOX* was up-regulated on Day 1 and rapidly down-regulated on Day 2. After removing the influence of light, *TPS* genes may be a key factor in regulating the diel rhythm of 3-Carene biosynthesis. 

## 3. Discussion

### 3.1. The Main VOCs of Oncidium Twinkle ‘Red Fantasy’ Were 3-Carene, Butyl Tiglate, and Prenyl Senecioate

Floral scent is one of the most important ornamental traits of plants, and is the main basis for evaluating flower quality. Compared with visual characteristics such as flower posture and color, the study of floral scent is relatively lagging behind. Due to the long life cycle and the complex and large genome of *Oncidium hybridum*, little was known about the synthesis and regulatory pathways of *Oncidium hybridum* floral scent. To date, the floral components of a variety of *Oncidium hybridum* have been identified. According to the types of floral scent component, terpenoids play a dominant role in the floral components of *Oncidium hybridum* [37]. Multiple studies have analyzed *Oncidium* Sharry Baby and identified its main volatile compounds as β-Ocimene, 3,7-dimethyl-1,3,6-Octatriene, Allo-ocimene, and 3-Carene, all of which belong to monoterpenes [32,38]. The main volatile compound of *Oncidium ornithorhynchum* and *Oncidium* Sweet Sugar was (*cis*)-3,7-dimethyl-1,3,6-Octatriene, while *Oncidium* ‘Wils. Golden Afternoon Rich Yellow’, *Oncidium*‘Gower Ramsey Gold 2’, *Oncidium* ‘Gower Ramsey’, and *Oncidium* ‘Rosy Sunset’ were 3,7-dimethyl-1,3,6-Octatriene, (-)-α-Cubebene, α-Copaene, and Linalool, respectively [37]. Monoterpenes are a class of important aromatic compounds, which are synthesized in plastids and are the main components of flower and fruit aromas [39]. We also identified 3-Carene, a monoterpene, as the main volatile component of *Oncidium* Twinkle ‘Red Fantasy’. 3-Carene is a bicyclic monoterpene that has a strong rosin flavor and is an important natural scent. In the *Dendrobium* genus of the Orchidaceae family, *Dendrobium chrysotoxum* and *Dendrobium hancockii* detected 3-Carene as the main aromas. 3-Carene has also been identified as one of the main aromatic compounds in *Oncidium* Sharry Baby and *Oncidium* ‘Red Fair’. Therefore, 3-Carene was the main component of the aroma compounds in *Oncidium* Twinkle ‘Red Fantasy’. 

In addition to terpenoids, two fatty acid derivatives (Butyl tiglate and Prenyl senecioate) were also identified in *Oncidium* Twinkle ‘Red Fantasy’. Fatty acid derivatives are another major class of compounds that make up the floral components of plants. In *Rosa hybrida*, the main component was geranyl acetate, which contributed to its unique aroma [18]. Methyl jasmonate was a class of fatty acid derivative and also the main aroma component of *Cymbidium ensifolium* [40]. Meanwhile, methyl benzoate was one of the most abundant odor compounds in snapdragon flowers [41]. Chen et al. [42] detected the aroma compounds of *Oncidium* Twinkle ‘Yellow Fantasy’ and *Oncidium* Twinkle ‘White Fantasy’. The results showed that 11 fatty acid derivatives were detected in *Oncidium* Twinkle ‘Yellow Fantasy’, including Isoamyl tiglate, Benzyl tiglate, 2-Methylbutyl acetate, Prenyl acetate, 2-Methylbutyl 2-methylbutyrate, 2-Methylbutyl isovalerate, Benzyl acetate, Methyl salicylate, Geranyl acetate, 2-methyl-butanoic aci phenylmethyl ester, and Benzyl valerate [42]. *Oncidium* Twinkle ‘White Fantasy’ detected five types of fatty acid derivative, including Benzyl tiglate, Methyl salicylate, Geranyl acetate, Methyl geranate, and Benzyl acetate [42]. Butyl tiglate and Prenyll senecioate were detected in *Oncidium* Twinkle ‘Red Fantasy’ of this study, but not in *Oncidium* Twinkle ‘Yellow Fantasy’ and *Oncidium* Twinkle ‘White Fantasy’. In summary, there were differences in the relative content of volatile compounds and floral composition among different species of *Oncidium hybridum*. 

### 3.2. Diurnal Rhythms of Aroma Synthesis Genes and VOCs Emissions in Oncidium Twinkle ‘Red Fantasy’ 

Floral scents also have important biological significance to the plants. Floral scents can not only resist the harm of pests and diseases to flowers, but also attract insects to help them complete the reproduction process. The release of floral compounds usually exhibits a clear diel rhythm, which was generally consistent with the corresponding pollination insect habits of plants. *Petunia hybrida* was pollinated mainly by moths and releases its main floral compounds at night, while the aroma compounds of snapdragon pollinated by bees were mainly released during the day [17,20]. In this study, the main floral compounds 3-Carene, Butyl tiglate, and Prenyl senecioate in *Oncidium* Twinkle ‘Red Fantasy’ were mainly released during the day, which may be related to the activity habits of pollinating insects such as bees. As early as 1729, De Mairan observed the circadian rhythm and the folding and unfolding leaves of a sensitive heliotrope plant in complete darkness, and suggested that these movements were related to the internal rhythms of the plant [43]. Henri-Louis Duhamel du Monceau and Alphonse de Candolle further demonstrated the existence of endogenous circadian rhythms, which were independent of the environment [44]. Plants have a biological clock that predicts seasonal changes and adjusts their physiology and development accordingly [45,46]. As an internal molecular timer, the biological clock measures periodic changes in the environment, including daily or seasonal fluctuations, and resets daily [43,45,46]. The circadian rhythm is endogenous, autonomous, and independent of environmental changes. Light is not the cause of circadian rhythms, but it can regulate and reset the phase of the circadian biological clock and synchronize it [44,47]. In petunias, the release of β-Ionone showed a consistent circadian rhythm. After 24 h of constant light treatment, the normal circadian rhythm was no longer displayed, which indicated that this phenomenon was regulated by the photoperiod [15]. The release of methyl jasmonate, a floral compound in *Cymbidium ensifolium*, exhibited a diel rhythm within 48 h (12 h light/12 h dark) [40]. Under 48 h of constant light treatment, the floral scent was not released [40]. Under 48 h of constant dark treatment, the release of scent from flowers has a rhythm, but the phase and amplitude of the rhythm have changed [40]. In our study, the release of 3-Carene, Butyl tiglate, and Prenyl senecioate exhibited the diel rhythm. 3-Carene maintained the diel rhythm under constant light/dark, but the phase and amplitude of its rhythm changed. Butyl tiglate and Prenyl senecioate showed the same release tendency under constant light/dark, and maintained a diel rhythm of photoperiod (12 h light/12 h dark). Therefore, the diel rhythm release of 3-Carene may be influenced by light and the circadian clock, and Butyl tiglate and Prenyl senecioate may be mainly determined by the endogenous circadian clock. 

Many physiological and biochemical activities of plants are regulated by genetic genes, and the release of floral compounds is closely related to gene expression. In the terpenoid and fatty acid derivative biosynthetic pathways, *DXPS*, *DXR*, *MCT*, *HDR*, *TPS*, *ATT*, and *LOX* genes have been shown to be associated with the diel rhythm of aroma release [17,18,23,24,25]. In *Oncidium* Twinkle ‘Red Fantasy’, we identified two *13-LOX* and six *9-LOX* genes in the biosynthesis pathway of fatty acid derivatives that exhibited a similar diel rhythm trend to the release of ester compounds. At the same time, 15 genes with the same trend as terpenoid release were identified, including *DXS*, *CMK*, *IDI*, and *TPS* genes. Therefore, the diel rhythm release of floral compounds in *Oncidium* Twinkle ‘Red Fantasy’ may be closely related to the expression of aroma synthesis-related genes. In general, the diel rhythm of aroma compounds was regulated by the circadian clock or photoperiod affecting gene expression. The circadian rhythm-related *bHLH* TF (*MYC2*) directly binds to the promoters of the terpenoid synthase genes *AtTPS11* and *AtTPS21*, and promotes their gene expression [48]. Zhou et al. [14] identified *MYB*-related TF *LATE ELONGATED HYPOCOTYL* (*JsLHY*) as a key gene regulating jasmine aroma formation, which can bind to the gene promoter regions of six aroma-related structural genes (*JsBEAT1*, *JsTPS34*, *JsCNL6*, *JsBPBT*, *JsAAAT5*, and *Js4CL7*) and directly promoted their expression. Yeh et al. [35] confirmed that CIRCADIAN CLOCK ASSOCIATED 1 (CCA1) in up-regulated *TPS* bound to the *TPS* promoter region, thereby affecting the circadian release of floral scent in *Oncidium* Sharry Baby. We identified 10 circadian rhythm-related TFs in the WGCNA network of aroma synthesis, including *TCP* (3), *MYB*-related (8), *bHLH* (1), and *bZIP* (1). Notably, eight *MYB*-related, one *bHLH*, and one *bZIP* genes predicted the regulation of the structural gene *Cluster-17371.14_TPS*, which was associated with terpenoid synthesis. Interestingly, the *TPS* genes were consistent with the release pattern of 3-Carene under constant dark treatment. This further indicated that *TPS* genes may play an important role in regulating the 3-Carene diel rhythm of *Oncidium* Twinkle ‘Red Fantasy’. 

## 4. Materials and Methods

### 4.1. Plant Materials

*Oncidium* Twinkle ‘Red Fantasy’ was planted in the Characteristic Orchid Germplasm Resources Preservation Nursery (Fuzhou, China). Before the experiment, 100 initial flowering plants were grown in a greenhouse at 30 °C/25 °C (14 h light/10 h dark). We selected *Oncidium* Twinkle ‘Red Fantasy’ at full flowering stage and cultured it at 25 °C for 12 h light/12 h dark (switching at 6:00 and 18:00) under constant light and constant dark treatment. The light intensity in this study was 20,000 Lx. Each treatment set up 30 plants to study the diel rhythm of VOCs emission and gene expression level. 

### 4.2. VOCs Collection and GC–MS Analysis

The headspace solid-phase micro-extraction (HS-SPME) technique coupled with the GC–MS system was used to collect and analyze *Oncidium* Twinkle ‘Red Fantasy’ floral volatile compounds, following the method described by Yeh et al. [35]. Samples were collected at 2:00, 6:00, 10:00, 14:00, 18:00, and 22:00 over 2 days, with a total of 12 time points. Three replicates were set for each sample. The whole floret organ was cut (1 g) and immediately sealed in a 20-mL headspace sample bottle, following the injection of 0.6 μL (240 μg/mL) *n*-nonane as an internal standard solution. The bottle equilibrated in a laboratory stirrer/hot plate at 40 °C for 10 min. Then, a stainless steel needle, housing 65 μm SPME fiber (Sigma Aldrich, St. Louis, MO, USA), was placed through the hole to expose the fiber at the position of 1 cm over the liquid surface for 30 min with magnetic stirring at 100 rpm. After extraction, the SPME device was inserted into the injection port of an Agilent 7890A gas chromatograph coupled with an Agilent 5975C (Agilent Technologies, Santa Clara, CA, USA) series mass spectrometer and desorbed at 250 °C for 5 min in a split/splitless GC injection port. The analytical conditions were as follows: the GC column was DB-5MS (30 m × 0.25 mm × 0.25 μm); the temperature of the injector and the MS interface temperature were set at 250 and 260 °C, respectively; the oven temperature programs of the DB-5MS columns were 35 °C (1 min), 2 °C/min to 80 °C (1 min), 5 °C/min to 160 °C (1 min), and 10 °C/min to 220 °C (10 min); the carrier gas was helium (99.9995%) at 1 mL/min; the injector mode was splitless; the mass spectrum in the electron impact mode was generated at 70 eV; and temperatures for ion source and the quadrupole mass filter were 230 and 150 °C. The identification of the component of floral scent used the MS spectra from NIST 17. The concentration was calculated using the formula C_voc_ = (A_voc_/A_std_) × (m_std_/m_s_), where m_std_ was the known level of internal standard and m_s_ was the weight of the sample; A_voc_ and A_std_ were quantifying ion peak areas of identified compound and internal standard, respectively; and C_voc_ was the concentration of the identified compound. 

### 4.3. RNA Extraction and Transcriptome Sequencing

In this study, RNA-seq sampling was performed at six time points (2:00, 6:00, 10:00, 14:00, 18:00, and 20:00) for 12 h light/12 h dark (switching at 6:00 and 18:00). Each sample was collected in three independent biological replicates. Total RNAs were isolated using ethanol precipitation and CTAB-PBIOZOL. The integrity and concentration of RNA were measured by Qubit fluorescence quantitative analyzer and Qsep400 high-throughput biological fragment analyzer, respectively. Illumina sequencing libraries were constructed and sequenced using the Illumina system NovaSeq6000. Due to the lack of a reference genome, we used Trinity (v2.13.2) [49] (https://github.com/trinityrnaseq/trinityrnaseq, accessed on 19 February 2024) to splice clean reads after obtaining them. --normalize_max_read_cov was used to set the maximum coverage of assembled reads (default: 50). --min_kmer_cov was used to set the minimum number of times (frequency) that k-mer must be observed. K-mers with frequencies below the threshold were excluded from the assembly (default: 5). --Min_gluemin number of reads needed to glue two inch contigs together (default: 10). We used the Corset (1.09) [50] (https://github.com/Oshlack/Corset, 19 February 2024) with default parameters compared to the expression of the number of reads on the transcript and model for hierarchical clustering transcript. We used Benchmarking Universal Single-Copy Orthologs (BUSCO) software (5.2.2) to evaluate the integrity of the assembled transcripts, and DIAMOND (v2.0.9) [51] software to compare the Unigene sequences with the Kyoto Encyclopedia of Genes and Genomes (KEGG), NCBI non-redundant proteins (NR), Swiss-Prot, gene ontology (GO), clusters of orthologous groups for eukaryotic complete genomes (KOG), and Trembl databases. We set the e-value to 1 × 10^−5^ to filter out results with poor alignment, and added the—max_hsps 1 parameter during alignment to only retain the hit that best aligned with the query sequence. After predicting the amino acid sequence of Unigene, HMMER software (3.1) (default parameter) was used to compare it with the Pfam database to obtain the annotation information of Unigenes. 

### 4.4. Analysis of RNA-Seq Data 

To estimate the expression levels of genes at different time points, clean transcriptome sequencing reads were aligned to the transcriptome obtained by Trinity splicing using Bowtie2 [52]. The number of reads mapped to each transcript was calculated using RNA sequencing by Expectation Maximization (RSEM) [53], and the fragments per kilobase of transcript per million mapped fragments (FPKM) was estimated to measure the transcript level of each gene [54]. DESeq2 (1.22.2) was used to analyze the difference of expression between sample groups. The Benjamini–Hochberg method was used to correct the hypothesis testing probability (*p*-value) by multiple hypothesis tests and obtain the False Discovery Rate (FDR). The screening criteria for differentially expressed genes (DEGs) were |log2Fold Change| >= 1 and FDR < 0.05. Subsequently, Gene Ontology (GO) [55,56] and the Kyoto Encyclopedia of Genes and Genomes (KEGG) [57] enrichment analysis were conducted for the DEGs. A pathway with FDR ≤ 0.05 was defined as one in which DEGs were significantly enriched. The gene expression values (FPKM) of all samples were analyzed by principal component analysis (PCA) linear algebra to evaluate the differences between groups and the situation of samples within groups. Related-genes expression heat maps were visualized using Tbtools (v2.119) [58]. We used iTAK (1.7a) software for transcription factor (TFs) prediction with default parameters. The WGCNA R software (4.2.1) package [59] was used for weighted gene co-expression network analysis (WGCNA), and a cluster tree was constructed based on the correlation between gene expression levels, and divided into modules. The threshold for module merging was set at 0.25, and the minimum number of module genes was 50. We visualized the co-expression network using Cytoscape (v3.5.1) software. 

### 4.5. RNA Extraction and qRT-PCR Analysis 

Total RNA samples were extracted with the FastPure Universal Plant Total RNA Isolation Kit (Vazyme, Nanjing, China), and cDNA synthesis was carried out according to the instruction manual of PrimeScript^TM^ II 1st Strand cDNA Synthesis Kit (Takara Bio, Kusatsu, Shiga, Japan). Finally, through using gene-specific primers (Table S1), RT-qPCR analysis was conducted by a QuantStudio 1 Plus system with 2X SYBR Green *Pro Taq* HS Premix (Rox Plus) (Accurate Biology, Changsha, China). The relative expression was calculated by the 2^−ΔΔCT^ method, and Actin was used as a reference gene to normalize the expression data. 

### 4.6. Statistical Analysis

Data were analysed using SPSS statistics (v.24). One-way analysis of variance (ANOVA) with Duncan tests was performed for multiple comparisons in this study. The steps in carrying out the ANOVA are: 1. Calculate the variance between the samples. To calculate the variance between the samples, take the sum of the squared deviations of the means of various samples from the grand average, and divide this total by the degree of freedom k-1, where k = number of samples. 2. Calculate the variance within samples. To calculate the variance within the samples, take the sum of squares of the deviations of various items from the mean values of the respective samples and divide this total by the degree of freedom, n-k, where n = total number of all the observations and k = number of samples. 3. Calculate the total variance. The total variation is calculated by taking the squared deviation of each item from the grand average and dividing this total by the degree of freedom, n-1, where n = total number of observations. 4. Calculate the F ratio. This measures the ratio of between-column variance and within-column variance. If there is a real difference between the groups, the variance between groups will be significantly larger than the variance within the groups. 5. Decision Rule. At a given level of significance, α = 0.05, and at n-k and k-1 degrees of freedom, the value of F is tabulated from the table. On comparing the values, if the calculated value is greater than the tabulated value, reject the null hypothesis. That means the test is significant or there is a significant difference between the sample means. The different letters in the histogram and line chart showed that there was a statistical difference in the mean value at *p* < 0.05. GraphPad (v.9.1.1) software was used to make expression graphs. 

## 5. Conclusions

**Figure 8:**
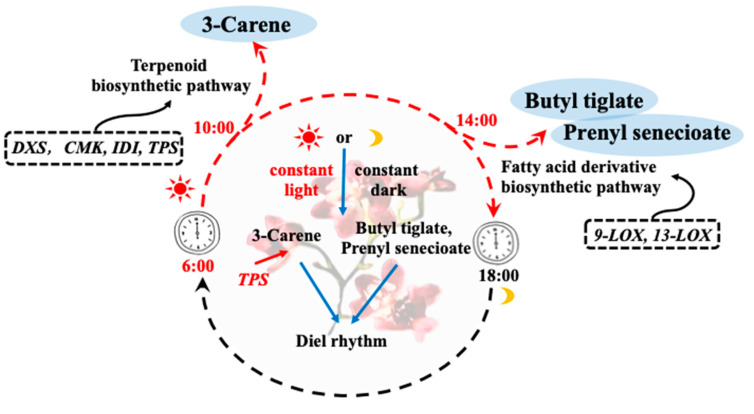
Schematic model of the mechanism by which the circadian rhythm regulates the aroma release of *Oncidium* Twinkle ‘Red Fantasy’. The main aroma compounds of *Oncidium* Twinkle ‘Red Fantasy’ were 3-Carene, Butyl tiglate, and Prenyl senecioate. 3-Carene were mainly released at 10:00, while Butyl tiglate and Prenyl senecioate were mainly released at 14:00. *DXS*, *CMK*, *IDI*, *TPS*, and *LOX* were key genes in the terpenoid or fatty acid derivative biosynthetic pathway, which were co-expressed with aroma release. Under the treatment of constant light or dark, the aroma release maintained a circadian rhythm.

In this study, volatile organic compounds and transcriptome analysis were used to clarify the mechanism related to the diel rhythm of aroma release in *Oncidium* Twinkle ‘Red Fantasy’. The main aroma compounds of *Oncidium* Twinkle ‘Red Fantasy’ were 3-Carene, Butyl tiglate, and Prenyl senecioate, and the aroma release had a diel rhythm. Through the constant light and constant dark treatments, it was further verified that the diel release of 3-Carene may be influenced by light and the circadian clock, and Butyl tiglate and Prenyl senecioate may be mainly determined by the endogenous circadian clock. Meanwhile, the *TPS* genes were consistent with the release pattern of 3-Carene, which may be a key factor in regulating the diel rhythm of 3-Carene biosynthesis (Figure 8).

## Data Availability

The datasets generated for this study can be found in the National Centre for Biotechnology Information (NCBI) Sequence Read Archive under accession number PRJNA1128565.

## Supplementary Materials

The following supporting information can be downloaded at: https://www.mdpi.com/article/10.3390/plants13223232/s1, Figure S1: total ion chromatogram of mass spectrometry analysis of *Oncidium* Twinkle ‘Red Fantasy’. ① Prenol; ② Nonane; ③ 3-Carene; ④ Butyl tiglate; ⑤ Prenyl senecioate. Figure S2: expression patterns of differentially expressed genes (DEGs) enriched in fatty acid derivative and terpenoid biosynthetic pathway. (a) Expression profiles of 21 DEGs enriched in fatty acid derivative biosynthetic pathway. (b) Expression profiles of 47 DEGs enriched in terpenoid biosynthetic pathway. Figure S3: the gene expression patterns of the Black, Green, Magenta, Pink, Purple, and Turquoise modules in WGCNA. The upper part was the clustering heatmap of genes within this module, with red indicating high expression and green indicating low expression. The lower part showed the expression patterns of module feature values in different samples. Figure S4: KEGG enrichment analysis of DEGs in modules. (a) KEGG enrichment analysis of DEGs in Blue module. (b) KEGG enrichment analysis of DEGs in Red module. (c) KEGG enrichment analysis of DEGs in Yellow module. (d) KEGG enrichment analysis of DEGs in Brown module. Table S1: primers used for real-time quantitative PCR.
