# Supplementary figures and images for: Emission and Transcriptional Regulation of Aroma Variation in Oncidium Twinkle ‘Red Fantasy’ Under Diel Rhythm
Source: Plants (Basel). 2024 Nov 17;13(22):3232. doi: 10.3390/plants13223232 (PMC11598538; doi:10.3390/plants13223232)

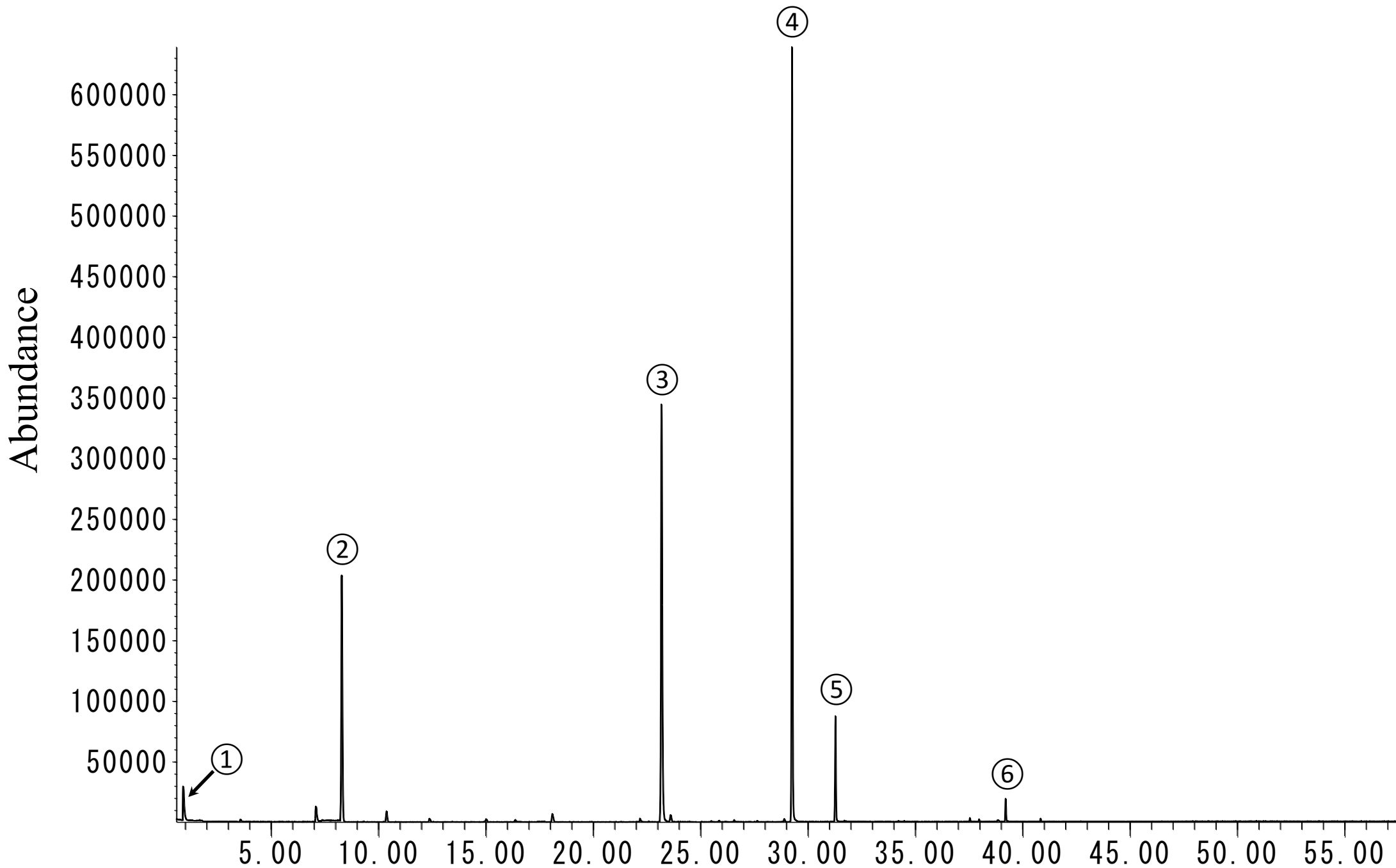

Supplement: Supplementary file 1 [file plants-13-03232-s001.zip › Figure S1.pdf]

(a)

## LOX pathway

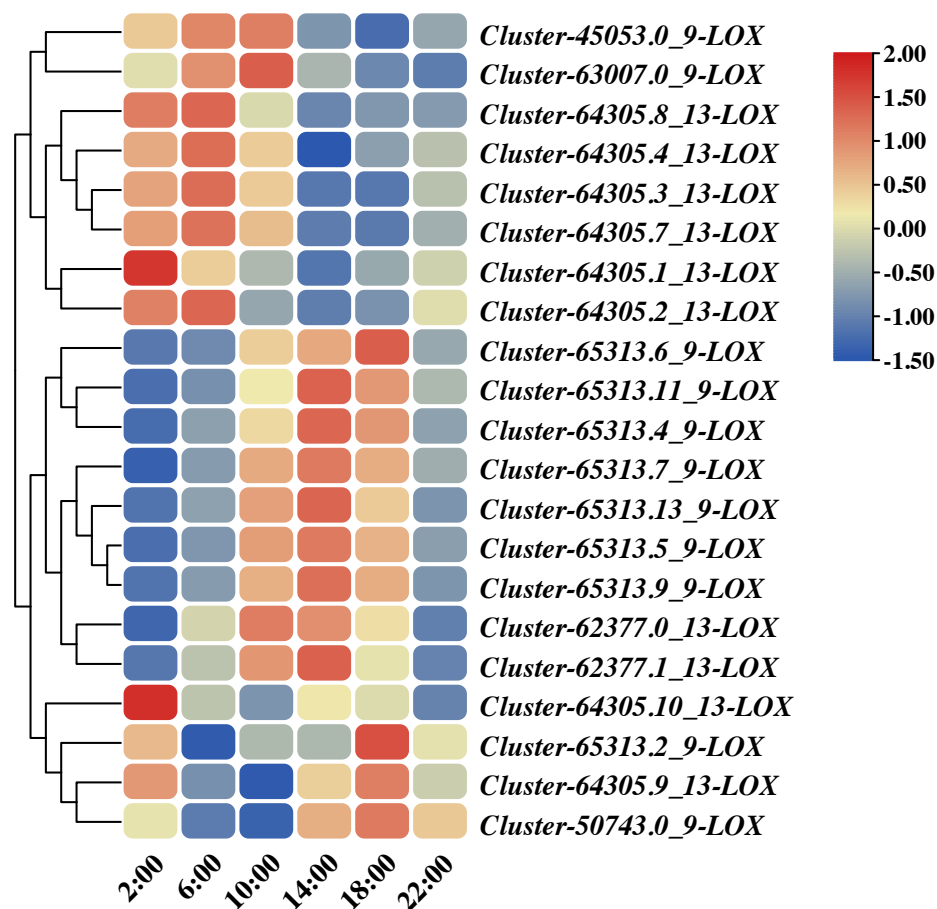

(b)

## MVA and MEP pathway

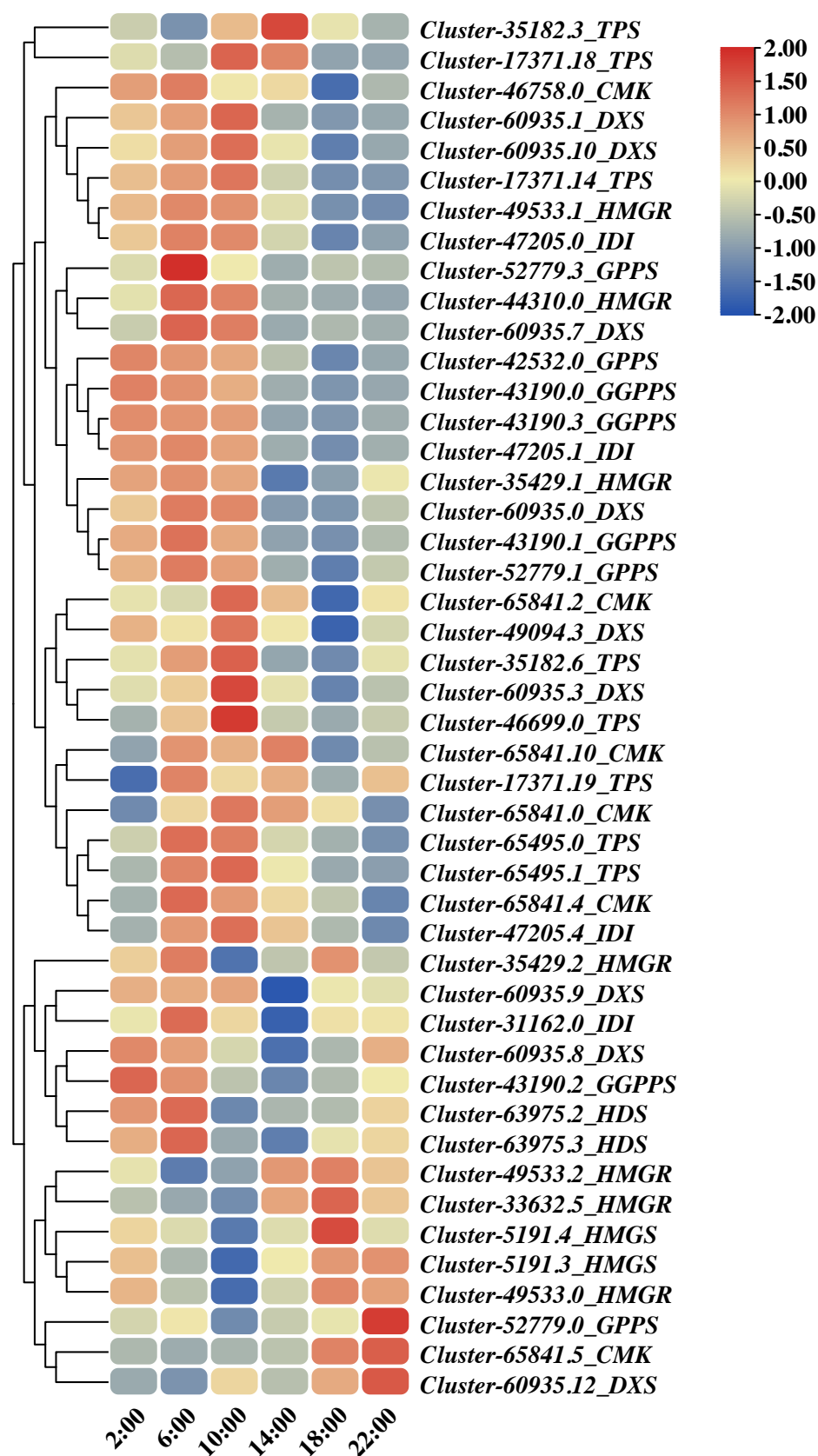

Supplement: Supplementary file 1 [file plants-13-03232-s001.zip › Figure S2.pdf]

Black module

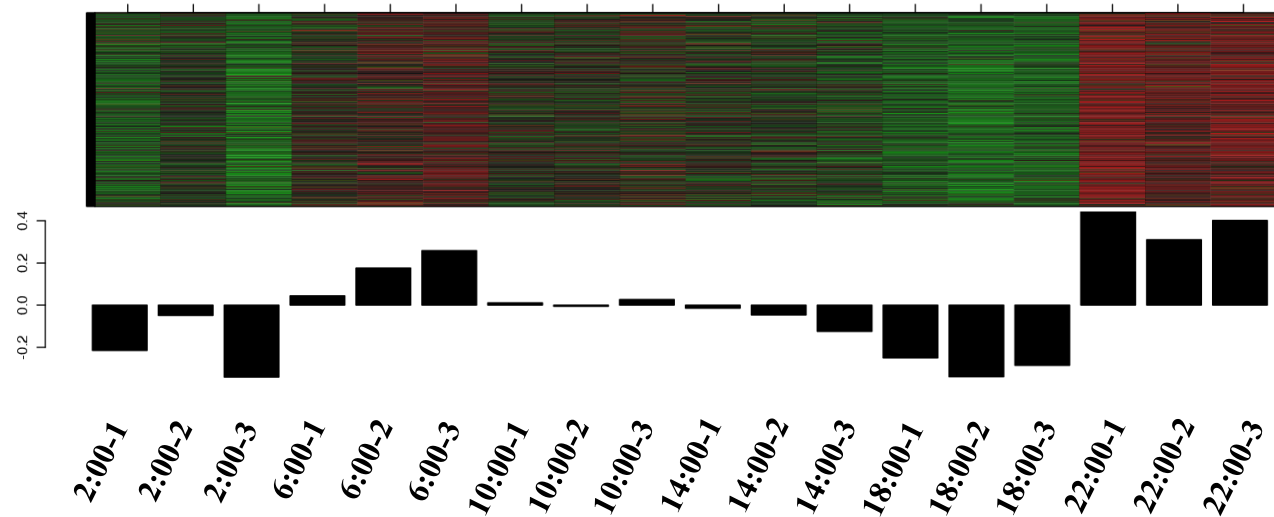

Green module

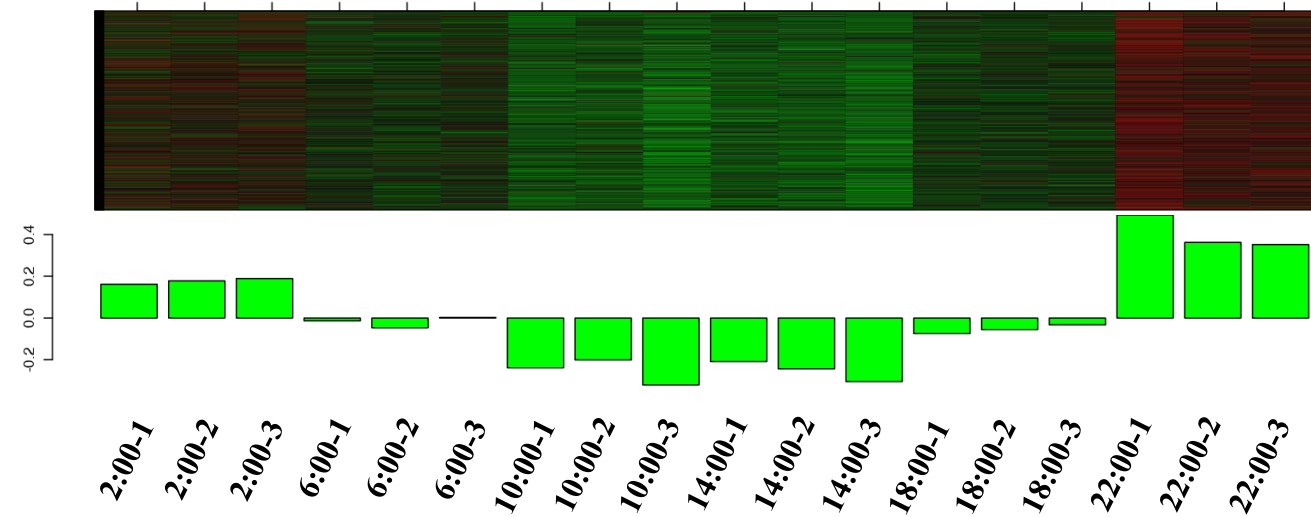

Magenta module

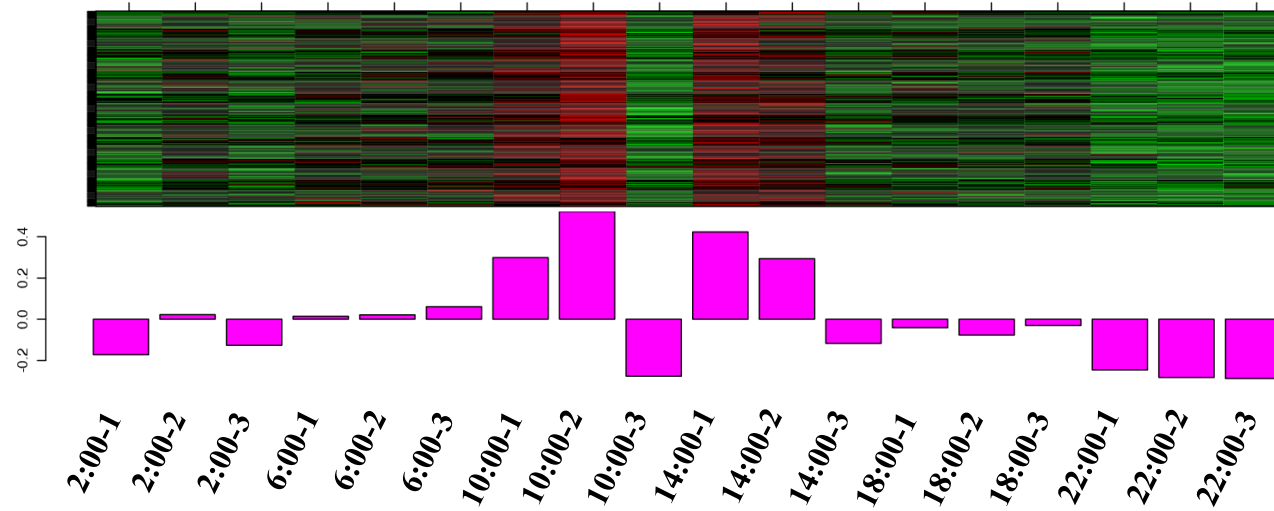

Pink module

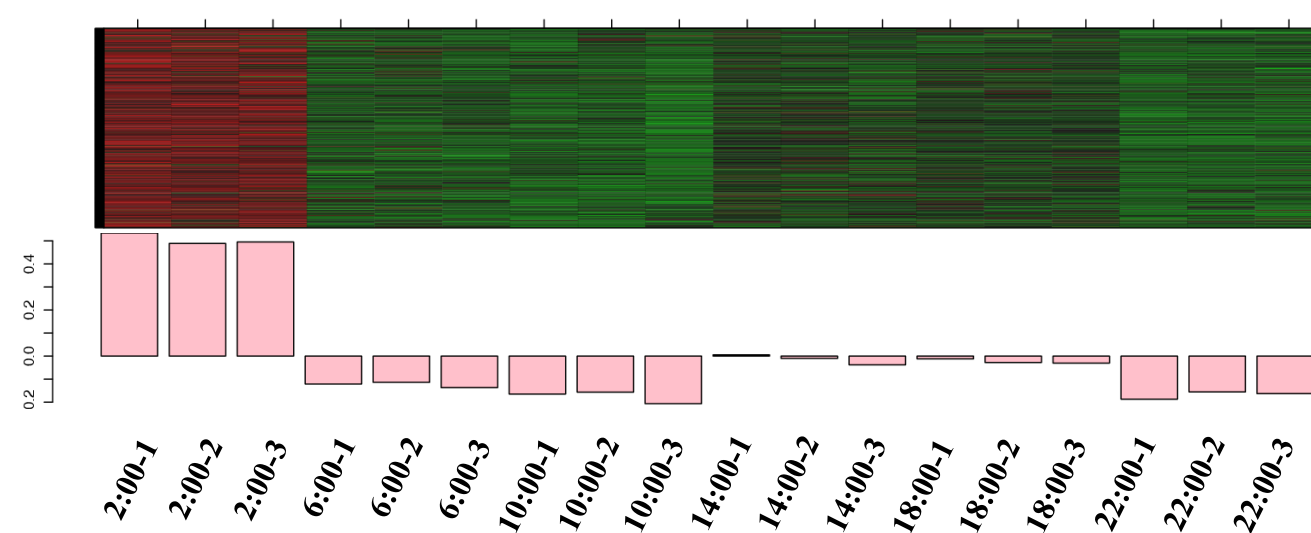

Purple module

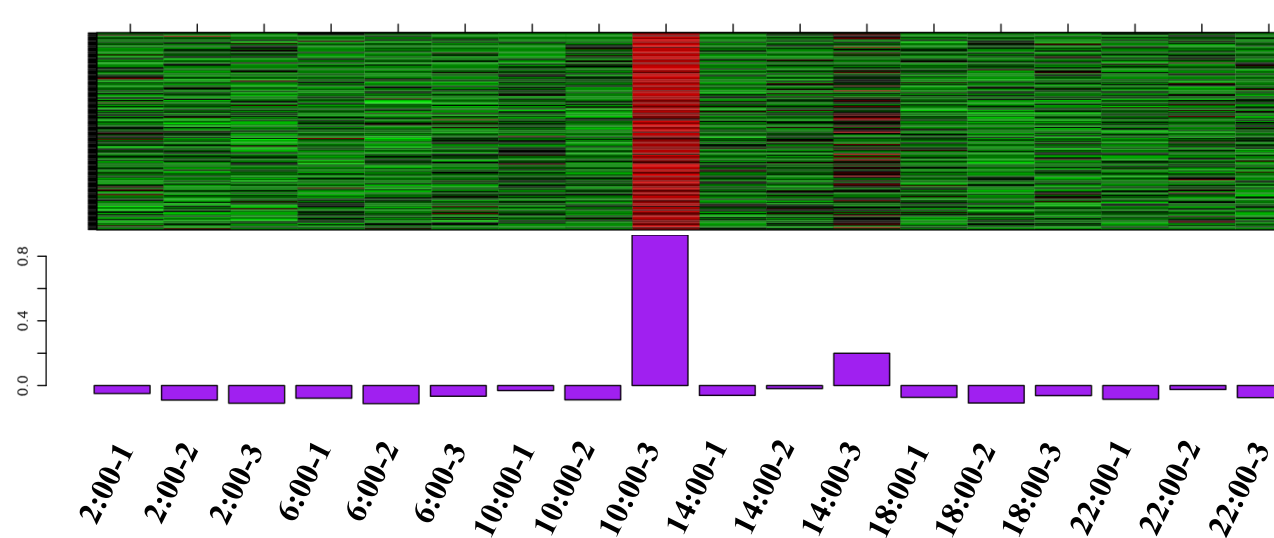

Turquoise module

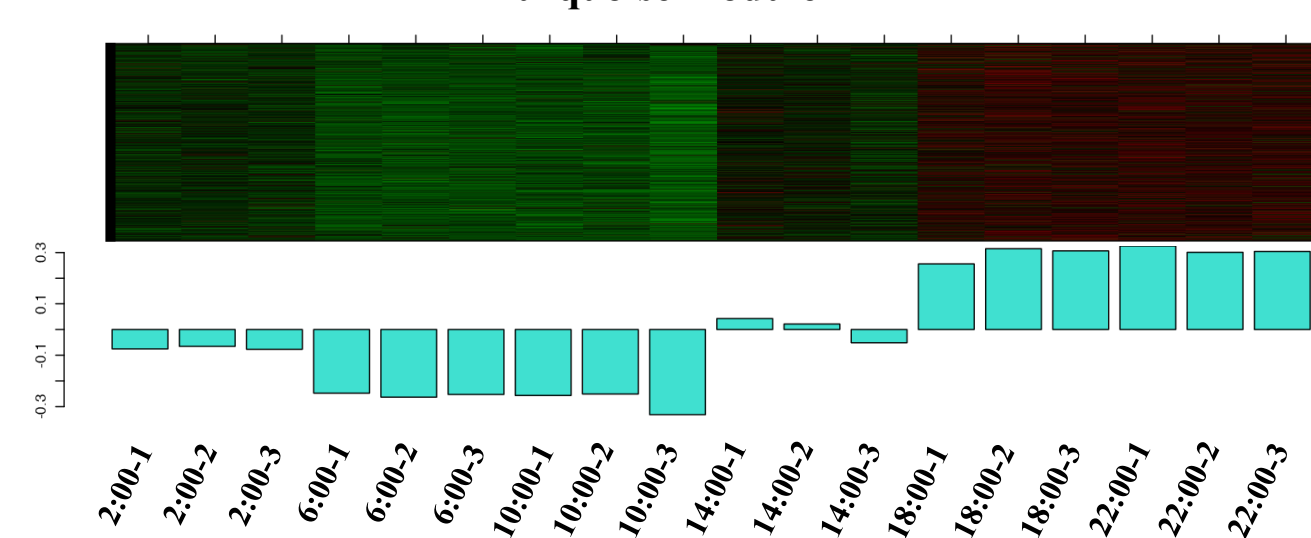

Supplement: Supplementary file 1 [file plants-13-03232-s001.zip › Figure S3.pdf]

(a)

## Blue module

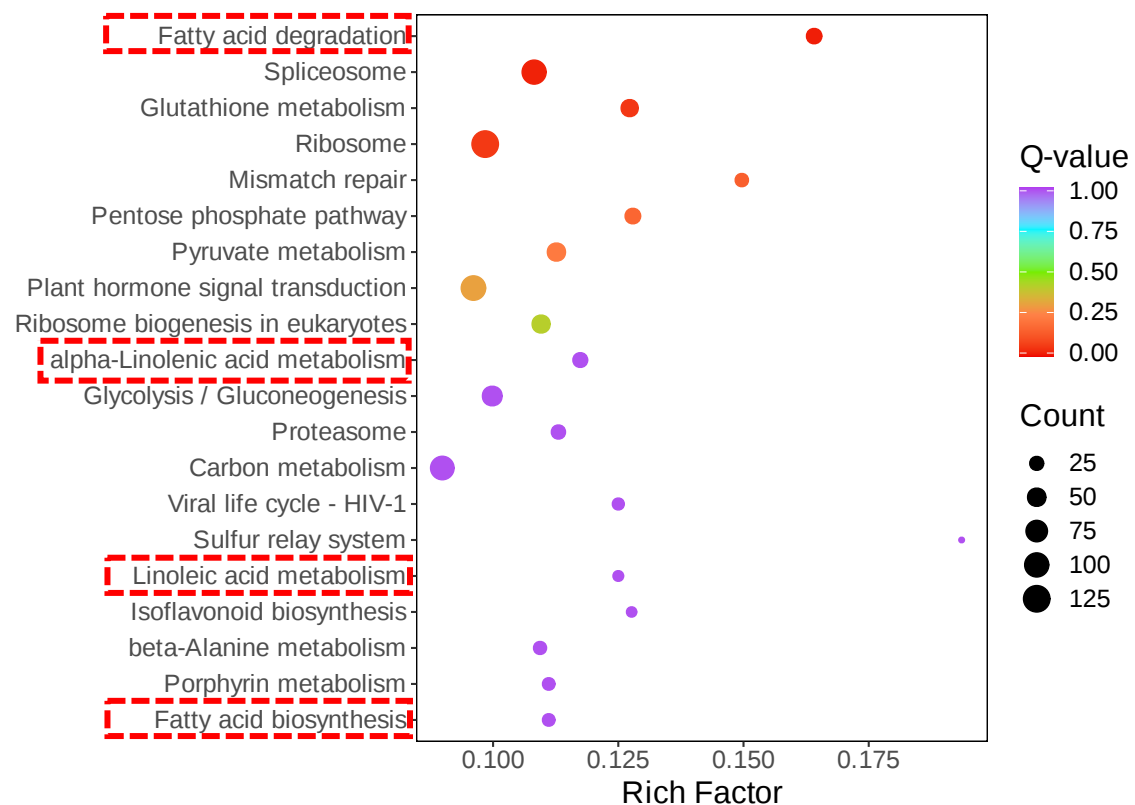

(b)

## Red module

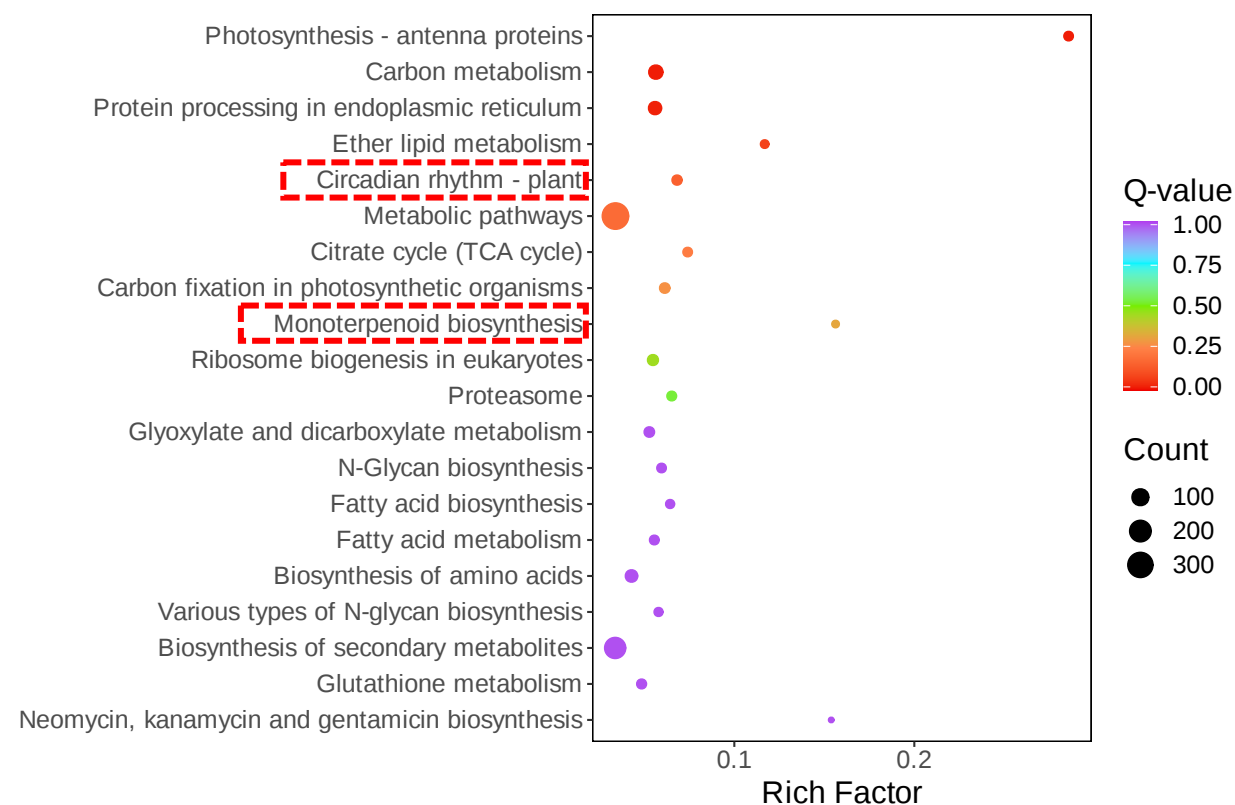

(c)

## Yellow module

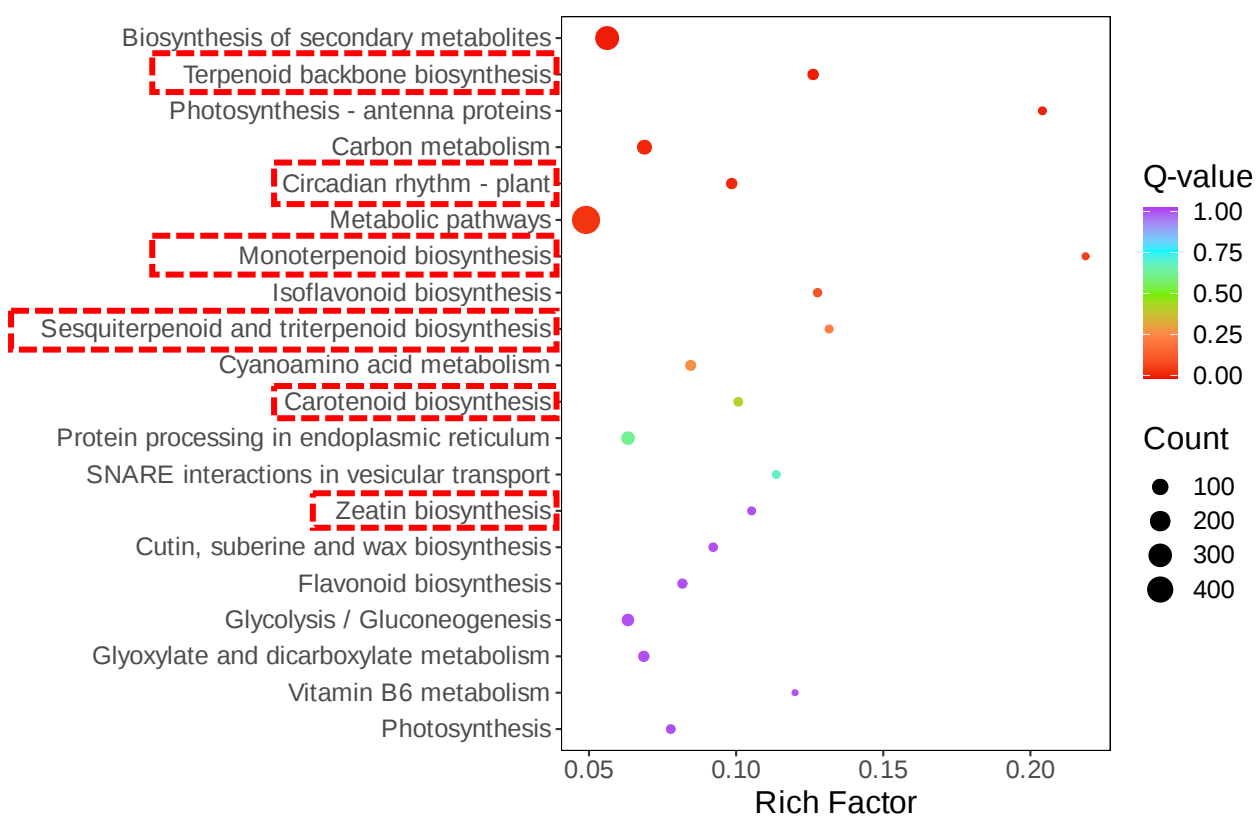

(d)

## Brown module

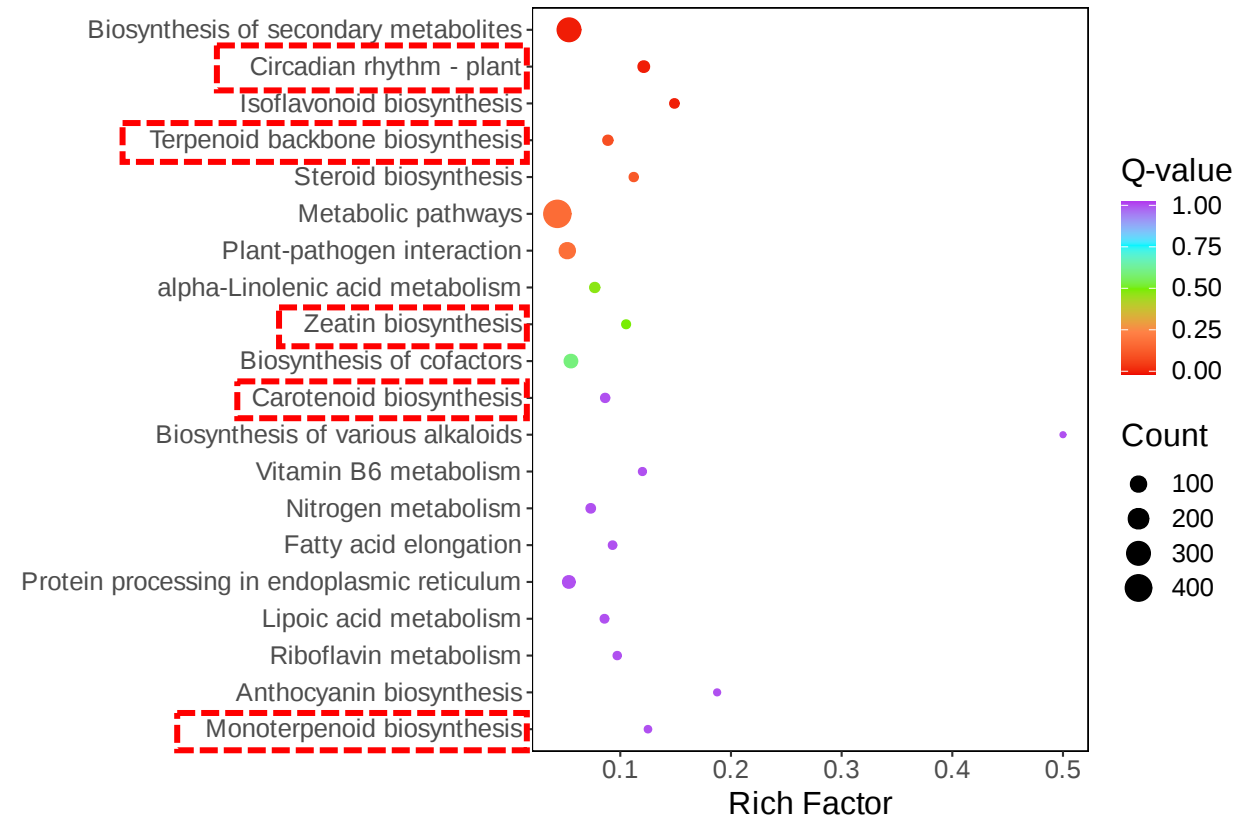

Supplement: Supplementary file 1 [file plants-13-03232-s001.zip › Figure S4.pdf]
